# Self-referencing versus other-referencing in gambling: effects of vmPFC stimulation on decision-making and feedback processing

**DOI:** 10.3389/fnbeh.2025.1634058

**Published:** 2025-09-09

**Authors:** Thomas Kroker, Maimu Alissa Rehbein, Miroslaw Wyczesany, Selina Hansen, Riccardo Bianco, Alejandro Espino-Paya, Markus Junghöfer

**Affiliations:** ^1^Institute for Biomagnetism and Biosignalanalysis, University of Münster, Münster, Germany; ^2^Otto Creutzfeldt Center for Cognitive and Behavioral Neuroscience, University of Münster, Münster, Germany; ^3^Unit of Clinical Psychology and Psychotherapy for Children and Adolescents, Institute of Psychology, University of Osnabrück, Osnabrück, Germany; ^4^Institute of Psychology, Jagiellonian University, Kraków, Poland; ^5^Department of Child and Adolescent Psychiatry, Psychosomatics and Psychotherapy, University Hospital Münster, Münster, Germany

**Keywords:** tDCS, vmPFC, MEG, affective learning, gambling

## Abstract

**Introduction:**

A key skill useful in everyday life is learning from our past choices to overcome cognitive biases and cope with our environment. In this regard, we are often responsible not only for ourselves but also for others.

**Methods:**

As our previous results showed that after excitatory stimulation of the ventromedial prefrontal cortex (vmPFC) people improved risk weighing and reduced their cognitive biases via improved affective learning, here we examined whether the above results differ when participants are playing for themselves versus for someone else. Therefore, we added this experimental manipulation to our previously well-validated gambling paradigm.

**Results:**

We found that participants showed improved learning after excitatory stimulation when playing for themselves but not when playing for someone else. At the neural level, we observed interaction effects involving the stimulation (inhibitory vs. excitatory), the frame (gain vs. loss) and the recipient (self vs. other) in prefrontal, temporal and parietal areas during the decision-making and feedback phase.

**Discussion:**

Our results suggest that excitatory vmPFC-tDCS can facilitate gambling and enhance the neural processing of gambling-related stimuli when playing for oneself.

## Introduction

We humans are characterized by the ability to think, refer and reflect on ourselves. Even more impressively, we can take the perspective of another person and infer about their thoughts, which is known as theory of mind (Carlson et al., 2013; Leslie et al., 2004). This enables us to understand and stand up for the interests of another person. However, there may be differences in how we pursue our own interests versus the interests of others.

As shown in functional magnetic resonance imaging (fMRI) studies, a core region for processing self-related information (first-person perspective, or self-referencing) is the ventromedial prefrontal cortex (vmPFC) (Jenkins et al., 2008; Soch et al., 2017; Stendardi et al., 2021). Indeed, while the activity of ventromedial prefrontal brain regions has been found to be correlated with an egocentric perspective, it has shown no such associations with an allocentric perspective (Vogeley and Fink, 2003). When processing information about others (third-person perspective, or other-referencing), different brain regions become active, such as the posterior cingulate cortex, precuneus and the dorsomedial prefrontal cortex (Soch et al., 2017). Yet, self-referencing seems to be a necessary condition for being able to take the perspective of another person (Jenkins et al., 2008), as has been revealed in hemodynamic neuroimaging studies and in patients with lesions in the medial prefrontal cortex (Jenkins et al., 2008; Soch et al., 2017; Stendardi et al., 2021; Vogeley and Fink, 2003).

Useful tools for studying the processing of self-related and other-related information in the brain include electroencephalography (EEG) and magnetoencephalography (MEG), as previous literature (Esslen et al., 2008; Knyazev, 2013) has shown that these tools can pick up effects in event-related potentials/fields (ERPs/ERFS), which provide high temporal resolution. Thus, these tools can complement the high spatial but low temporal resolution of hemodynamic methods. From studies using these tools, differences between self-referencing and other-referencing conditions were found to occur in early components after 130 ms, but also in later event-related potential components such as N2 and P3, with P3 being described as the most prominent. The latest ERP components showing differences between the processing of self-and other-related information emerged between 400 and 500 ms (Esslen et al., 2008; Knyazev, 2013). Consistent with the above fMRI results, EEG findings have also suggested that ventromedial prefrontal areas are particularly important for self-referencing, while referencing to others has been shown to activate more dorsal prefrontal areas (Bradford et al., 2019; Gallagher and Frith, 2003). These neural differences were also reflected in behavioral differences in the perception of oneself and others (Bradford et al., 2019); specifically, participants were faster and more accurate in processing information about themselves compared to processing information about others (Bradford et al., 2019). Thus, these findings not only highlight the differences in processing information about oneself versus another, but they indicate that both neural and behavioral dimensions should be examined.

Another valuable tool for investigating brain function is transcranial direct current stimulation (tDCS), which allows brain activity to be modulated from outside the skull (Nitsche et al., 2008; Sparing and Mottaghy, 2008). This allows causal inferences to be made through non-invasive stimulation with little or no side effects (Poreisz et al., 2007). For stimulating the vmPFC, tDCS provides advantages to repetitive transcranial magnetic stimulation (rTMS), as it does not induce uncomfortable co-stimulation of the eye muscles.

In the current study, we opted to examine differences in self-referential versus other-referential information processing by combining prior findings indicating that the vmPFC is involved in self-referencing with our previous findings from gambling paradigms. In prior studies, we showed that in a gambling situation, excitatory stimulation of the vmPFC induced enhanced affective learning, leading to reduced cognitive biases and increased overall gains compared to sham and inhibitory stimulation (Kroker et al., 2022, 2023a, 2023b, 2025). In these prior studies, we have also been particularly interested in the so-called framing effect, whereby we humans are strongly influenced by how an option is presented (i.e., “framed”). For example, in a previous study we gave participants 50 cents to gamble with and presented two options, namely the “gamble” option and the “keep” option. Here, the “keep” option could be presented in one of two ways: it could be framed as a gain or a loss. In the gain-frame condition participants were presented the option of keeping 20 of the 50 cents, and in the loss-frame condition participants were presented the option of losing 30 cents (i.e., 50 to 30 cents). Participants tended to gamble more often in the loss-frame condition, which is irrational because both options have the same value, namely an outcome of +20 cents. Accordingly, the smaller the difference between the gain frame minus the loss frame in risk-taking behavior and in the outcome ratings, the more rationally the participants behaved. Interestingly, this “framing difference” became smaller after excitatory compared to sham and inhibitory stimulation of the vmPFC (Kroker et al., 2022, 2023a, 2023b). While in the previous studies participants were always gambling for themselves (egocentric perspective), in the current study we asked participants to gamble for themselves in half of the trials but for someone else in the other half of the trials. In this way, here we investigated whether risk weighing, learning and the framing effect differed between conditions of self-and other-referencing, whether these effects were modulated by stimulation and whether both factors revealed any kind of interaction.

By combining the well-known fact that the vmPFC is a hub for self-referencing (Bradford et al., 2019; Soch et al., 2017) with findings from our previous studies, we derived our current hypotheses. In the decision-making phase, we expect to replicate a reduced framing effect at the behavioral and neural level after excitatory vmPFC stimulation, which should be particularly pronounced when playing for oneself. Furthermore, we anticipate observing an enhanced processing depth of stimuli after excitatory stimulation in the self-referencing condition, also on the behavioral level, which will be reflected in improved learning over the course of the experiment after excitatory vmPFC stimulation, as observed in our previous studies (Kroker et al., 2023a, 2023b, 2025), but this improvement should be stronger in the self-referencing compared to the other-referencing condition. At the neural level, we anticipate an interaction effect of stimulation and recipient, featuring enhanced activity of ventral prefrontal areas in response to trials in which the participants gamble for themselves after excitatory stimulation but with no effect of stimulation in the other-referencing condition.

In the feedback phase (i.e., with presentation of win or loss feedback), we expect attenuated behavioral biases (i.e., a reduced framing effect reflecting more rational gambling) on the Self-Assessment Manikin (SAM-ratings) (Bradley and Lang, 1994) after excitatory stimulation when participants play for themselves. Furthermore, we expect neural correlates of these reduced framing effects after excitatory stimulation within medial prefrontal areas, as research suggests that these areas are associated with the framing effect in gambling (DeMartino et al., 2006; Kroker et al., 2022, 2023b) as well as self-and other-referencing (Bradford et al., 2019; Jenkins et al., 2008; Vogeley and Fink, 2003).

## Methods

### Participants

We included 32 (17 female) right-handed volunteers aged 19–29 years (*M* = 23.42, *SD* = 2.70). Exclusion criteria were current or lifetime psychiatric diagnosis, psychopharmacological treatment, current or past psychotherapy, neurological or severe somatic illness, pregnancy and prior participation in one of the previous gambling studies in our lab (the latter would result in knowledge about our cover story). Participants were recruited from our institute’s existing participant pools, announcements on the university campus and via social media.

Participants were pseudo-randomly assigned to one of the two experimental groups of tDCS stimulation (see below), which were matched for demographic and psychometric characteristics. The study was ethically approved by the Ethics Committee of the Medical Faculty of the University of Münster. All research was conducted in accordance with the Declaration of Helsinki.

Participants were told a cover story to ensure authentic gambling behavior. They were informed that, in addition to the fixed amount of €30, they could win an amount between €0 and €36 for themselves, and they would also have the opportunity to win money for others. Additionally, they were told that any money they earned for others would be evenly distributed among the other participants of the study. At the end of the study, participants were informed about the cover story, and everyone received the maximal amount of €66 for themselves. For details, please consult the Supplementary material.

### Experimental procedure

**Figure 1:**
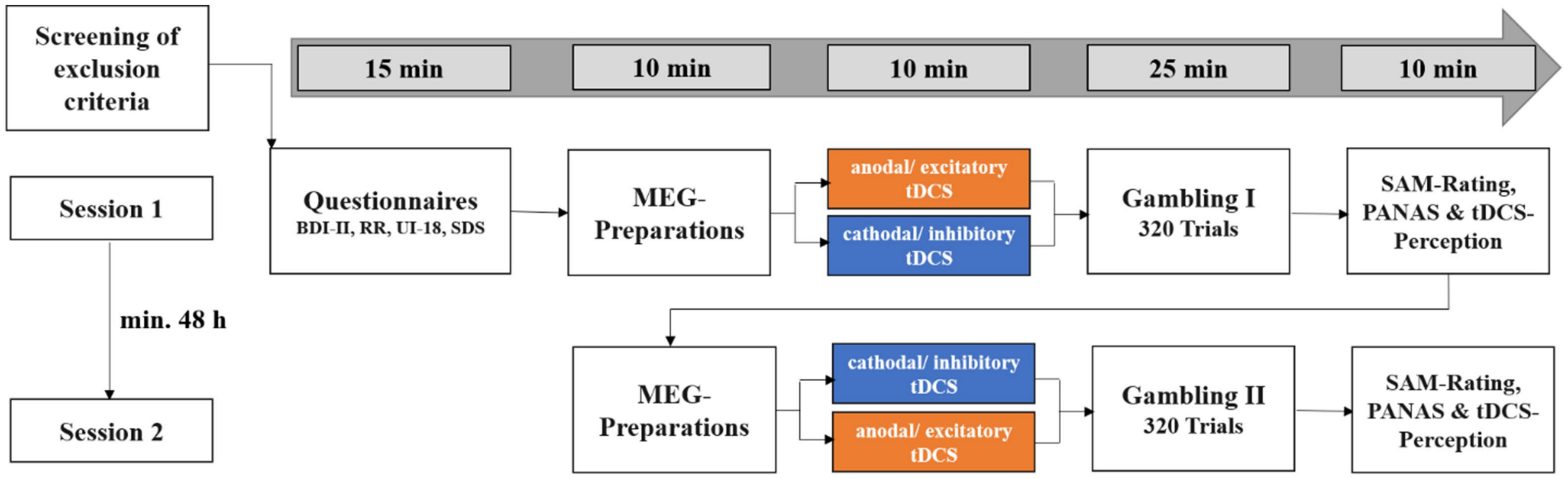
Overview of the experimental procedure. BDI-II, Beck Depression Inventory-II; RR, Reward Responsiveness Scale; UI-18, Intolerance of Uncertainty Scale; SDS-CM, Social Desirability Scale by Crowne and Marlowe; SAM-Rating, subjective ratings of hedonic valence and emotional arousal; PANAS, Positive and Negative Affect Schedule. This figure was published first by Kroker et al. (2022). Excitatory and inhibitory stimulation did not differ in perceived pleasantness and intensity (*p*-values >0.150).

**Figure 2:**
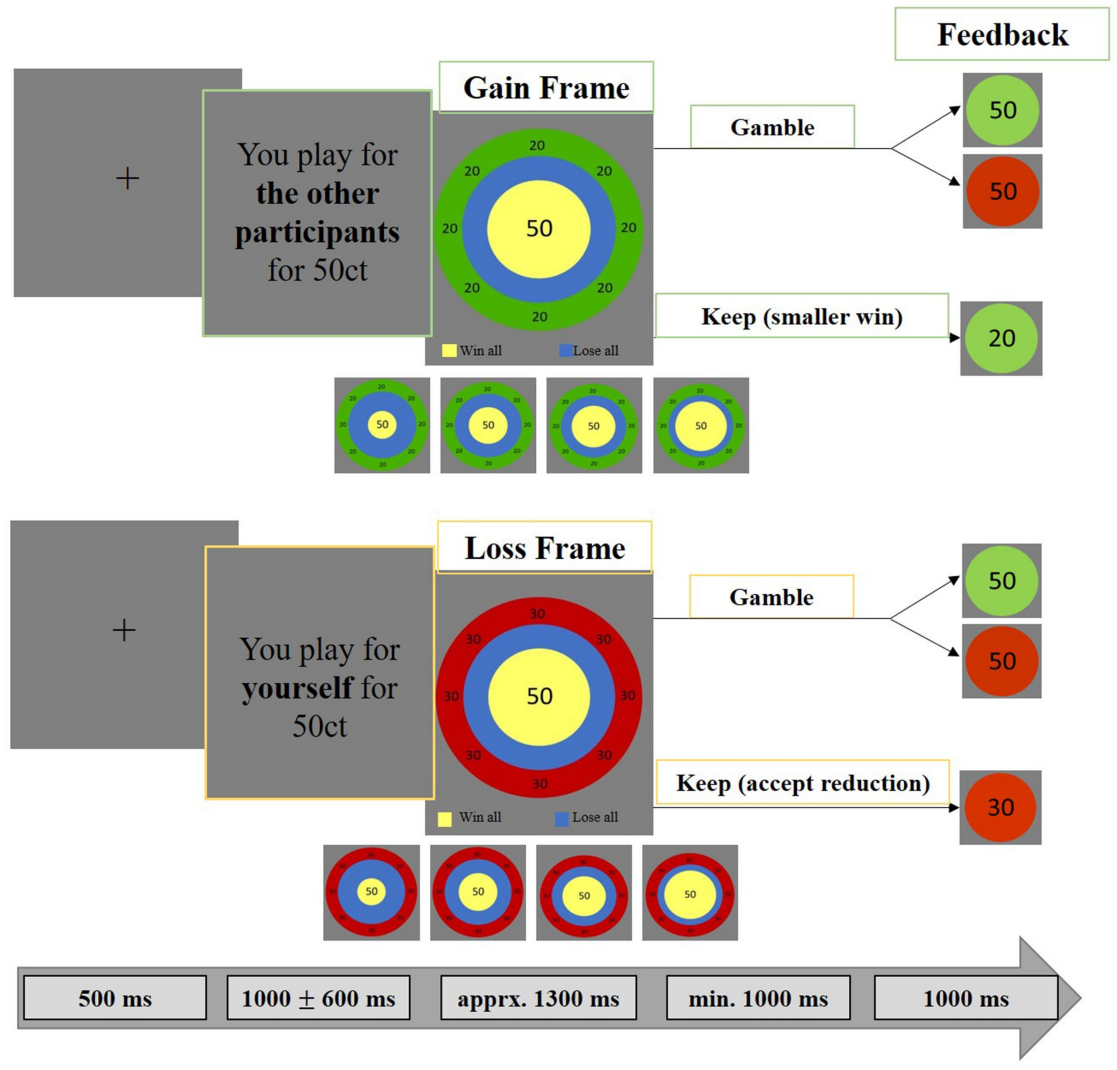
Monetary gambling task. The paradigm consisted of a choice and a feedback phase. Stimuli were placed centrally to minimize eye movements and related MEG artifacts. Each trial began with a 500 ms fixation cross, followed by the “game stake” (25, 50, 75, or 100 cents) and the recipient (self, indicated in yellow or others, indicated in green). Participants then chose to either secure a smaller amount (“keep”) or risk the full amount (“gamble”). The “keep” option was framed as a gain (safe win) or loss (safe loss). Risk levels (20, 40, 60, 80%) altered expected values, making “keep” preferable at 80% risk and “gamble” preferable at 20 and 40% risk, while at 60% risk both options had equal expected value. We analyzed effects of vmPFC stimulation and self-/other-referencing on gambling behavior and MEG correlates of neural activity evoked by the choice and feedback stimuli.

In a within-subjects design, each participant received excitatory and inhibitory stimulation over the course of two experimental sessions, with a minimum interval of 48 h and a maximum of 30 days between sessions (see Figure 1). Participants were informed of the two appointments and that they would receive two stimulations. However, they were not told that we were not using a placebo condition in order to avoid possible inferences about the experimental hypotheses. The order of stimulation (excitatory or inhibitory stimulation first) was randomized across participants. At the beginning of the first session, participants gave written informed consent then filled in questionnaires comprising the Beck Depression Inventory (Beck et al., 1996), the Reward Responsiveness Scale (Van den Berg et al., 2010), the Intolerance of Uncertainty Scale (Gerlach et al., 2008) and the Social Desirability Scale (Crowne and Marlowe, 1960). We did not expect responses to these questionnaires to be altered by the stimulation, as they reflect stable personality traits. Following the stimulation, participants performed the gambling task in the MEG, where event-related fields (ERFs) were measured in response to the choice and feedback stimuli. At the end of each session, participants rated the feedback in terms of subjective hedonic valence and emotional arousal on a Self-Assessment Manikin (SAM) rating scale (Bradley and Lang, 1994), rated their mood on the Positive and Negative Affect Schedule (PANAS; Watson et al., 1988) and rated the perceived pleasantness of the stimulation and the stimulation intensity on an in-house questionnaire. In the second session, the same procedure was used with the opposite stimulation polarity (see Figure 2). Finally, subjects were informed about the cover story. They were informed about the stimulation conditions and which stimulation was conducted when. The total duration of the two sessions was approximately 200 min.

### Gambling task

Each trial began with a fixation cross presented for 500 ms, followed by the presentation of the “game stake” of 25, 50, 75 or 100 cents and who they were playing for (others, self; see Figure 1). Second, the “choice stimulus” appeared, on the basis of which participants had to decide whether they wanted to secure a smaller amount (“keep”) or risk the full amount (“gamble”). The “keep” option was framed as either a gain (gain frame: receiving a smaller but safe amount) or as a loss (loss frame: losing a smaller safe amount). The framing effect is relevant to the “keep” option: for example, if the stake is 50 cents (Figure 1), the green gain frame communicates a sure win of 20 cents (i.e., equivalent to a sure loss of 30 cents), while the red loss frame predicts a sure loss of 30 cents (i.e., equivalent to a safe win of 20 cents). According to this scheme, for initial amounts of 25ct, 75ct or 100ct, the gain frames inform about safe wins of 10ct, 30ct or 40ct, respectively, and the loss frames inform about safe losses of 15ct, 45ct or 60ct, respectively. In addition to varying the recipient (others, self), the frame (gain frame, loss frame) and the initial amount (25ct, 50ct, 75ct, 100ct), we also varied the risk of losing (20, 40, 60, 80%). Importantly, this “risk of losing” variable resulted in different expected values for the “keep” and the “gamble” option, so that it is more adaptive to choose “keep” in the 80% risk condition and to choose “gamble” in the 20 and 40% risk conditions. In the 60% risk condition, “gamble” and “keep” had the same expected value.

### tDCS

Transcranial direct current stimulation (tDCS) is a widely used and effective method for non-invasively modulating brain activity from outside the skull. Anodal or excitatory stimulation depolarizes the membrane potential of neurons, which increases their excitability depending on the strength of the applied electric field. In contrast, cathodal or inhibitory stimulation hyperpolarizes the neural membrane, reducing the likelihood of action potentials (Sparing and Mottaghy, 2008). For simplicity and readability, we will refer to excitatory and inhibitory stimulation instead of anodal/cathodal stimulation. An important advantage of tDCS is the low rate of side effects (e.g., headache, nausea and insomnia) and, with particular relevance for ventral prefrontal target regions, the absence of unwanted co-stimulation of facial or ocular muscles and nerves. Changes in cortical excitability can persist for up to an hour after a single stimulation (Poreisz et al., 2007).

**Figure 3:**
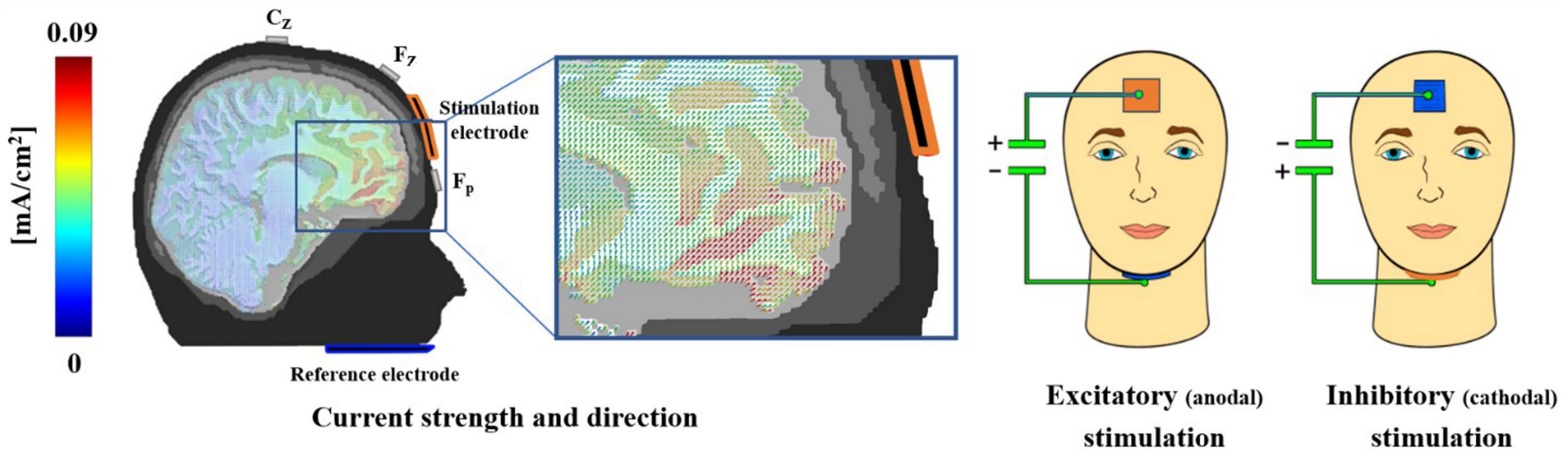
An iterative gain function algorithm aimed at maximum vmPFC-targeted stimulation resulted in the use of a small mid-frontal electrode and an extended extracephalic chin reference. This array allowed for quasi-reference-free stimulation, offering clear differentiation of excitatory and inhibitory effects. Participants were stimulated on two different days for 10 min at 1.5 mA in either an excitatory (anodal forehead electrode) or inhibitory (cathodal forehead electrode) manner. While the current strength was identical for excitatory and inhibitory stimulation, the direction of effect, as indicated by the cones in the magnification, was reversed. A modeled 1.5 mA stimulation resulted in a maximum current density in the vmPFC regions of approximately 0.09 mA/cm^2^ (red colors). In the experiment, all sponges were the same color to avoid any inferences from participants based on the sponge color. This figure was published first in Junghöfer et al. (2017).

We implemented the tDCS montage as used in our previous studies to stimulate the vmPFC (Junghöfer et al., 2017; Kroker et al., 2022, 2023a; Rehbein et al., 2023; Roesmann et al., 2021; Winker et al., 2018, 2019, 2020). The active electrode was placed on the forehead (3 × 3 cm), and the extraencephalic reference, allowing for a quasi-reference-free stimulation, was placed under the chin (5 × 5 cm). The electrodes were inserted into sponges soaked in a sodium chloride solution to ensure electrical conductivity. For excitatory or inhibitory stimulation, the forehead electrode was used as the anode or cathode, respectively. This electrode configuration results in maximal stimulation of the vmPFC and minimal stimulation of adjacent brain regions, as shown by finite-element-based forward modeling of tDCS currents (Wagner et al., 2014). Using a DC Stimulator Plus (NeuroConn GmbH), we administered a maximum current of 1.5 mA for 10 min with both stimulation polarities (see Figure 3).

### Recording and preprocessing of MEG

Event-related fields were measured with a 275 whole-head sensor system (CTF Systems, first-order axial gradiometers) at a sampling rate of 600 Hz over a frequency range of 0 to 150 Hz (hardware anti-aliasing filtering). The continuous data were downsampled to 300 Hz and filtered with a 0.1 high-pass filter and a 48 Hz low-pass filter. Individual head shapes were measured using a 3D tracking device (Polhemus, Colchester, VT, United States; http://www.polhemus.com/) and individual head positions in the MEG were gathered by three landmark coils (i.e., fiducials) in the ears and on the nasion. We extracted epochs from 200 ms before and 600 ms after stimulus onset and used the interval from −150 ms to 0 ms for baseline fitting. To identify and reject artifacts, we used the method proposed by Junghöfer et al. (2000). This method detects individual and global artifacts: when noisy channels are identified, their signal is estimated by spherical-spline interpolation based on the weighted signal of all remaining sensors. On average two channels were interpolated per subject. A minimum threshold of 0.01 was applied to the estimated goodness of interpolation, and trials exceeding this value were rejected (on average 18 of 320 trials per subject). If more than 30% of the trials in a session were discarded, the participant was excluded from further analysis (eight participants). This resulted in a final sample for the neural data of 24 participants. Trials within each experimental condition were averaged for each participant and session, and the underlying neural sources of the measured ERFs were estimated by applying L2 minimum-norm estimates (Hämäläinen and Ilmoniemi, 1994) (L2-MNE). The L2-MNE is an inverse modeling technique that estimates the underlying neuronal sources based on the measured magnetic fields. A spherical model comprising 350 dipole pairs distributed evenly in both the azimuthal and polar directions, with a source shell radius corresponding approximately to the gray matter depth (i.e., 87% of the individually fitted head), was employed as the source model. The topographies of the L2-MNE were established using a Tikhonov regularization parameter of *k* = 0.1. Source-direction-independent neural activities (i.e., the vector length of the estimated source activities at each position) were then calculated for each participant, condition, and time point. Due to the inverse problem, MEG (as EEG) cannot differentiate between neural activity evoked by deeper more focal sources from weaker but more superficial and distributed sources as both may evoke identical MEG signals. With realistic head models and application of L2-Minimum-Norm depth weighting, this inverse problem can be somewhat attenuated. As individual MRIs for realistic source modeling was not available, the L2-Minimum-Norm was applied without depth weighting. Thus, potential neural activity generated by deeper neural structures are projected to the surface and superpose with neural activity evoked by sources in the estimated gray matter depth. Individual noise covariance estimation, which could lead to improved precision in source reconstruction, was also not applied. The preprocessing and analysis of the MEG data was performed using the MATLAB (2023a)-based EMEGS software (Peyk et al., 2011) (version 3.1).

### Data analysis

We used mixed-effects models for the behavioral data because of their robustness with repeated measures compared to conventional models (Baayen et al., 2008). Following our previous studies (Kroker et al., 2022, 2023a, 2023b), we calculated the same analysis but added the factor recipient (others, self). For the behavioral analyses, we used the full sample of 32 participants. All results remained qualitatively equivalent when only the MEG sample was used.

Thus, for behavioral analyses, we performed a mixed-effects logistic regression with the predictors stimulation (excitatory, inhibitory), frame (gain frame, loss frame) and recipient (others, self) to analyze binary choice behavior (i.e., “gamble” or “keep”). Additionally, we calculated a further mixed-effects logistic regression with the predictors stimulation (excitatory, inhibitory), risk of losing (20, 40, 60, 80%) and recipient (others, self). Since we also investigated improved affective learning from gambling stimuli in our previous studies, we were interested in how learning would interact with the recipient. Therefore, we calculated another mixed-effects logistic regression with the predictors stimulation (excitatory, inhibitory), risk of losing (20, 40, 60, 80%), recipient (self, others) and trial number (1–320). In these analyses, we modeled random effects for stimulation. Furthermore, we performed control analysis by including the factor stimulation order to check for potential carryover effects. To analyze the perceived hedonic valence and arousal SAM-rating (Bradley and Lang, 1994) of the feedback (nine-point Likert scale), we computed a mixed-effects linear regression with the predictors stimulation (excitatory, inhibitory), decision (keep, gamble), outcome (gain, loss) and recipient (others, self). Since we were particularly interested in the framing effect, we conducted a separate analysis only in the “keep” condition, because here the gain and loss frames have the same value, just the framing is different. To this end, we calculated the difference between the gain-and loss-framed ratings, since the larger the framing difference, the more irrational the rating.

For the neural analyses, we computed repeated measures ANOVAs and corrected for multiple comparisons using a non-parametric approach proposed by Maris and Oostenveld (2007). The two analyses in the decision-making phase (stimulation by frame by recipient and stimulation by risk-to-lose by recipient) were performed separately from each other to ensure a sufficient number of trials per condition for a reasonable signal-to-noise ratio in the source estimation. To analyze the neural data of the feedback-processing phase, we calculated 2 × 2 × 2 × 2 repeated measures ANOVA with the factors stimulation (excitatory, inhibitory), decision (keep, gamble), outcome (gain, loss) and recipient (others, self). As with the behavioral data, we performed an analysis only in the framing-relevant “keep” condition and calculated the framing difference. Since we were mainly interested in main or interaction effects with stimulation, only these effects are depicted here in the main text. All resulting clusters were then tested for main or interaction effects involving stimulation order, as in the behavioral data. Demographic variables, such as gender, and psychometric measures such as mood were not included as covariates in the main analyses because preliminary checks revealed no significant associations with the dependent variables. In addition, we had no specific hypothesis regarding this measures. Including them as covariates would have unnecessarily reduced statistical power and added model complexity without improving explanatory value. Further effects are shown in the Supplementary material.

## Results

### Decision-making

**Figure 4:**
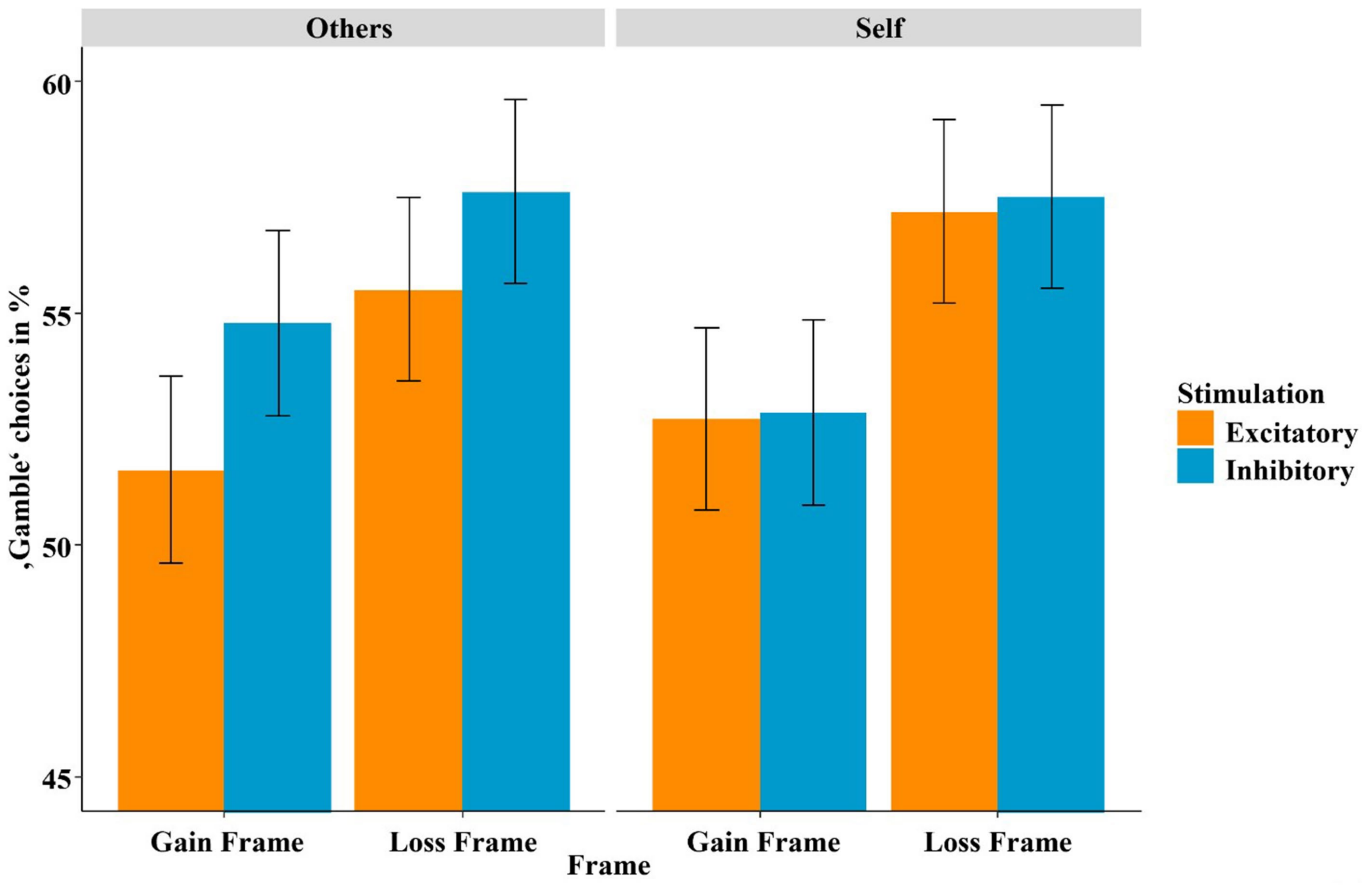
Percentage of “gamble” choices (*y*-axis) for gain-framed and loss-framed trials (*x*-axis). An “ideal rational agent” would have chosen the gain-framed option and the loss-framed option with equal frequency, as both resulted in identical wins or losses. However, replicating a strong deviation from rationality due to loss aversion, participants more often chose the risky “gamble” option in the loss-framed condition. Importantly, replicating our previous findings, participants gambled more often after inhibitory compared to after excitatory vmPFC stimulation. Bars indicate mean and 95% confidence intervals.

The mixed-effects logistic regression using the predictors stimulation (excitatory, inhibitory), frame (gain frame, loss frame) and recipient (others, self) yielded an overall significant model [*χ*^2^(9) = 197.32, *p* < 0.001], and within this model, the predicted main effect of frame became significant: (*z* = 2.94, *p* = 0.003, OR = 1.20). As expected, independent of the recipient, participants chose the gamble option more often in response to the loss frame (see Figure 4). Additionally, the main effect of stimulation (*z* = −2.05, *p* = 0.040, OR = 1.11) reached significance, indicating that, again as predicted, participants gambled more often after inhibitory compared to after excitatory vmPFC stimulation. The predicted interaction effect of stimulation by frame, with stronger effects of stimulation in the loss frame, which we have repeatedly shown in previous studies (Kroker et al., 2022, 2023a, 2023b), was insignificant (*z* = 0.75, *p* = 0.457), as was the three-way interaction of stimulation by frame by recipient (*z* = 0.790, *p* = 0.430). The control analysis including stimulation order revealed no main or interaction effect involving stimulation (all *p*-values >0.2, all *t*’s ± <1.2).

**Figure 5:**
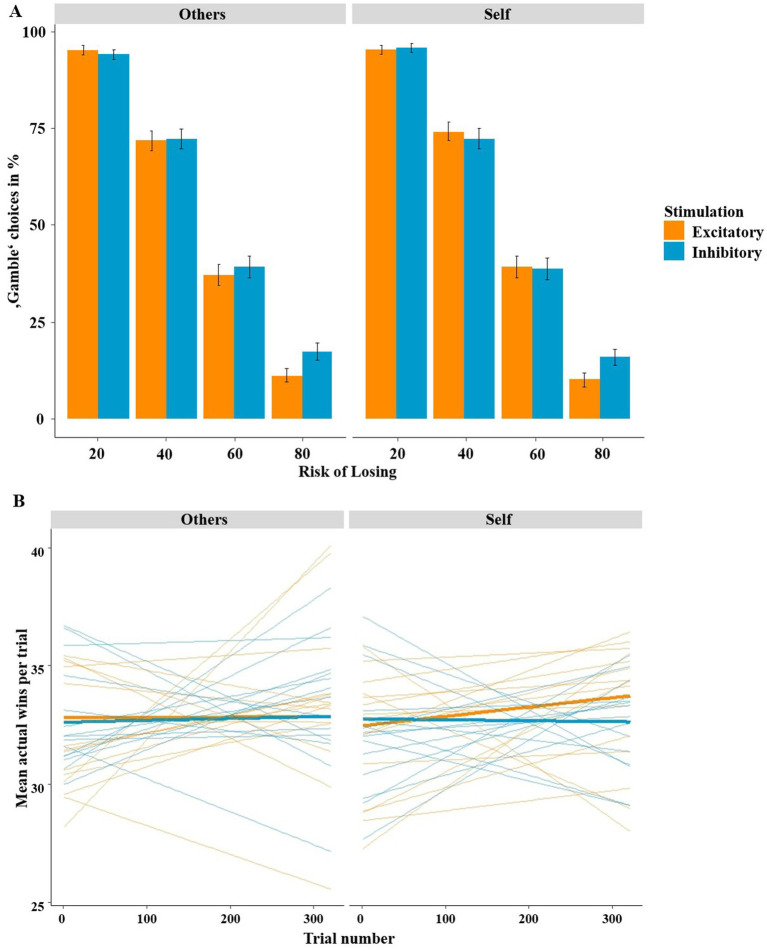
**(A)** Percentage of “gamble” choices (*y*-axis) according to the risk (*x*-axis) and recipient (self vs. other). After excitatory compared to inhibitory stimulation, participants tended to gamble more adaptively/advantageously, as they gambled more often in the high chance of winning conditions (20 and 40% risk) and less often at the high risk of losing condition (80% risk). This effect of stimulation occurred for both self-and other-referencing conditions alike. Bars indicate mean and 95% confidence intervals. **(B)** Mean actual wins per trial (*y*-axis) according to the trial number (*x*-axis) stimulation and recipient (self vs. other). Learning gradients clearly indicate that participants showed improved affective learning after excitatory compared to after inhibitory stimulation when playing for oneself but not when playing for others. Averaged regressions within stimulation groups are shown with bold lines, individual regressions with thin lines.

The further mixed-effects logistic regression employing the predictors stimulation (excitatory, inhibitory), risk of losing (20, 40, 60, 80%) and recipient (others, self) also showed a significant overall model [*χ*^2^(9) = 9714.00, *p* < 0.001]. Here, the unsurprising main effect of risk of losing was significant, as participants preferred the safe “keep” option and gambled less when the risk of losing was high (*z* = −38.32, *p* < 0.001, OR = 0.12; see Figure 5A). Importantly, we were able to replicate the hypothesized adaptive/advantageous effect of stimulation on the risk of losing (stimulation by risk of losing: *z* = 3.60, *p* = 0.003, OR = 1.30). In fact, after excitatory stimulation, participants chose the “gamble” option more often when the chance of winning was high and opted for the “keep” option more often when the risk of losing was high, as shown in our preceding studies. This adaptive effect of stimulation was independent of the factor recipient, as the three-way interaction of stimulation by risk of losing by recipient was not significant (*z* = −0.294, *p* = 0.787). The control analysis including the factor stimulation order revealed an interaction of stimulation, risk of losing and stimulation order (*z* = 4.55, *p* < 0.001, OR = 1.99). However, none of the effects were involving the recipient (*z*-values < ± 1.2, *p-*values > 0.25). The respective figures were shown in the Supplementary material.

Finally, the mixed-effects logistic regression with the predictors stimulation (excitatory, inhibitory), risk of losing (20, 40, 60, 80%), recipient (others, self) and trial number (1–320) also showed a significant overall model [*χ*^2^(17) = 9714.00, *p* < 0.001]. As in our previous studies, the three-way interaction of stimulation by risk of losing by trial number was significant (*z* = 3.64, *p* < 0.001, OR = 1.33; see Figure 5B), indicating improved learning after excitatory compared to after inhibitory stimulation. However, and interestingly, this predicted three-way interaction was modulated by the factor recipient (*z* = −2.37, *p* = 0.017, OR = 0.88). *Post hoc* tests revealed that improved affective learning after excitatory stimulation occurred only when participants played for themselves, as the three-way interaction of stimulation by risk of losing by trial number was significant within trials where participants played for themselves (*z* = 3.28, *p* = 0.001, OR = 1.24) but not when participants played for others (*z* = 1.27, *p* = 0.203). This led to increasing wins per trial after excitatory stimulation in the “self” conditions over time (see Figure 5B), whereas no increase in wins across trials were observed in the “others” conditions (i.e., excitatory other, inhibitory other, inhibitory self). As in the previous analysis the stimulation order had a significant impact on the learning pattern (*z* = −3.42, *p* < 0.001, OR = 0.61). Again, the effects did not involve recipient (*z*-values < ± 0.6, *p-*values >0.5).

**Figure 6:**
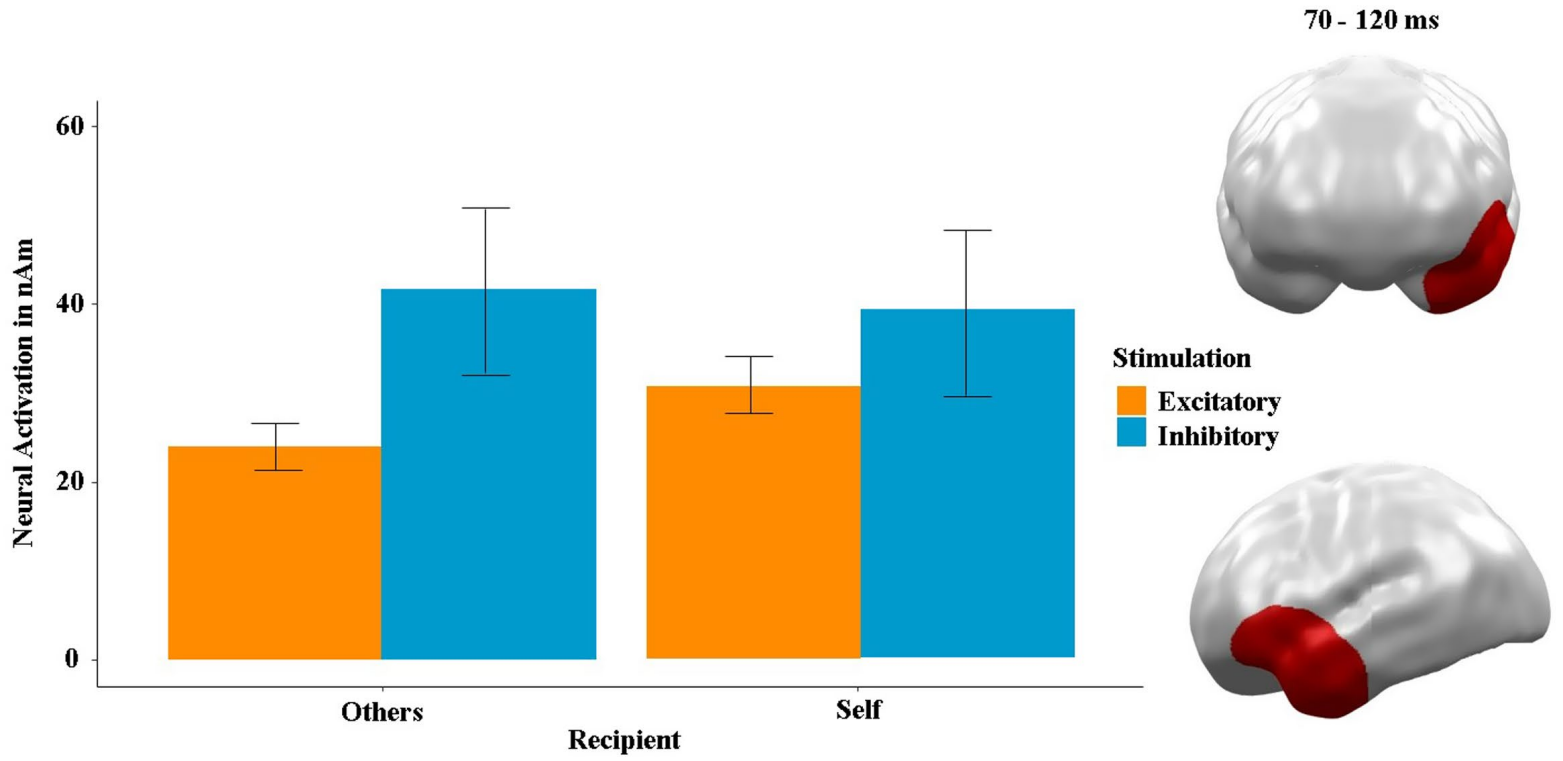
Significant spatiotemporal cluster in left orbitofrontal prefrontal and anterior temporal areas featuring an interaction of stimulation by recipient (others vs. self). Bars indicate mean and 95% confidence intervals. Topographies of effects observed in L2-MNE were projected on standard 3D brain models for visualization.

To investigate the neural basis of this behavioral finding, we examined the interaction effects of stimulation, rational decision-making and recipient in the MEG data (see Figure 6). We found a significant neural spatiotemporal cluster for the interaction of stimulation by recipient between 70 and 120 ms in the left orbitofrontal and anterior temporal areas (*p-*cluster = 0.045). *Post hoc t*-tests showed that the difference between stimulations was significant within the “others” condition [*t*(23) = −2.74, *p* = 0.012, *η*^2^ = 0.15] but not within the “self” condition (*t* = −1.47, *p* = 0.143). Furthermore, the neural activity in response to the “self” condition was significantly higher after excitatory stimulation (*t* = −2.56, *p* = 0.024, *η*^2^ = 0.13), while this comparison was insignificant following inhibitory stimulation (*t* = 0.18, *p* = 0.854). Interestingly, the cluster was negatively correlated with gambling (i.e., risky) choices (*ρ* = −0.18, *p* = 0.014) after excitatory stimulation and positively correlated with gambling choices (*ρ* = 0.19, *p* = 0.010) after inhibitory stimulation. The correlation was inverted not only between the stimulations but also between the recipients (others: *ρ* = −0.42, *p* < 0.001; self: *ρ* = 0.35, *p* < 0.001). No neural cluster showed any main or interaction effect involving stimulation order (all *p*-values >0.3, all *t*’s ± <1.4).

No other main or interaction effect involving the factor stimulation was significant in the MEG data of the decision phase.

### Feedback processing

**Figure 7:**
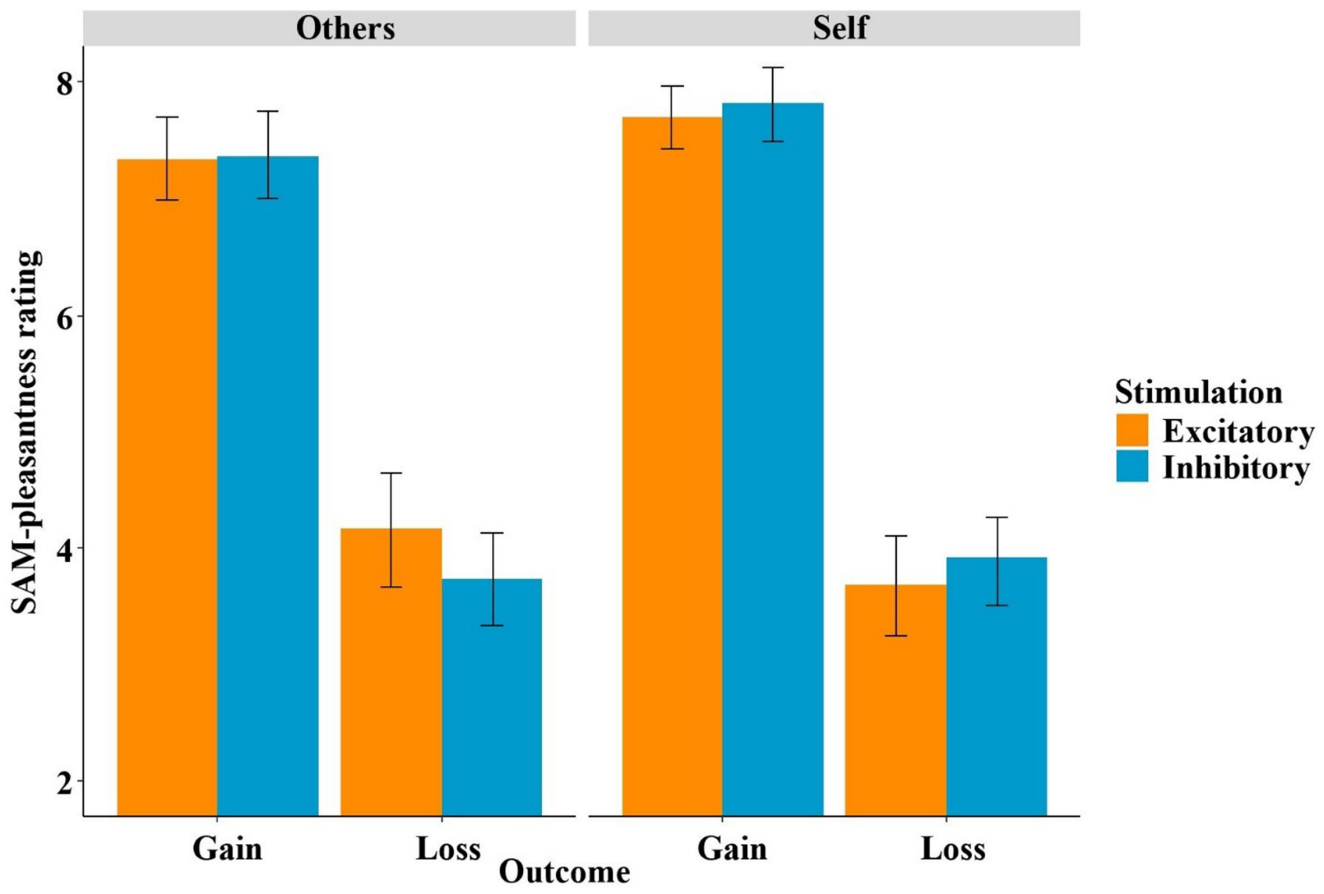
SAM-pleasantness rating of feedback as a function of stimulation, outcome and recipient. In particular, the stimulation modulated the way participants processed losses depending on the recipient (self vs. other).

In the SAM-pleasantness feedback ratings, the mixed-effects linear regression using the predictors stimulation (excitatory, inhibitory), decision (keep, gamble), outcome (gain, loss) and recipient (others, self) to predict the SAM-pleasantness rating resulted in an overall significant model [*χ*^2^(17) = 514.91, *p* < 0.001]. Within this model, the unsurprising main effect of outcome became significant (*t* = −14.66, *p* < 0.001, *η*^2^ = 0.88), as gains were rated more positively than losses (see Figure 7). Furthermore, the interaction of stimulation by recipient (*t* = 2.14, *p* = 0.042, *η*^2^ = 0.14) and the three-way interaction of stimulation by outcome by recipient (*t* = 2.05, *p* = 0.048, *η*^2^ = 0.13), and, thus two interaction effects involving the factor stimulation, became significant. The three-way interaction was mainly driven by the two-way interaction of stimulation by recipient in the loss condition (*t* = 2.29, *p* = 0.028, *η*^2^ = 0.15), while the respective interaction in the gain condition remained insignificant (*t* = 0.41, *p* = 0.68). Within the “others” and the “loss” conditions, the difference between the stimulation conditions was trend significant (*t* = 1.53, *p* = 0.090, *η*^2^ = 0.03), while in “self” and “gain” conditions, the difference between the stimulation conditions was not significant (*t* = −0.78, *p* = 0.217).

With our special interest in the framing effect, we also calculated a model within the framing-relevant “keep” condition only employing the predictors stimulation (excitatory, inhibitory), outcome (gain, loss, here: frame) and recipient (others, self). In this model, no main or interaction effect involving stimulation was significant (all *p*-values >0.3, all *t*’s ± <1.4). This revealed a significant difference between gain-and loss-framed outcomes (*t* = 11.09, *p* < 0.001, *η*^2^ = 0.81) even though they held the same value, indicating a strong framing effect. In addition, the two-way interaction of outcome (frame) by recipient was significant (*t* = −2.12, *p* = 0.041, *η*^2^ = 0.11). To illustrate the framing effect more clearly, we calculated the difference between the gain frame and the loss frame (i.e., gain frame minus loss frame), which indicated a greater framing difference when playing for yourself than when playing for others (*t* = −2.32, *p* = 0.033, *η*^2^ = 0.12).

Regarding the SAM-arousal feedback rating, we used the same four predictors (stimulation, decision, outcome and recipient), which again revealed a significant model [*χ*^2^(17) = 150.87, *p* < 0.001]. Unsurprisingly, the main effect of decision was significant (*t* = −7.48, *p* < 0.001, *η*^2^ = 0.66), indicating higher arousal ratings after “gamble” choices compared to the safe outcomes in the “keep” option. Additionally, the main effect of recipient became significant (*t* = 2.33, *p* = 0.027, *η*^2^ = 0.16), with higher arousal ratings when participants played for themselves. The main effect of outcome was insignificant (*t* = −0.48, *p* = 0.630). However, the interaction effect of stimulation by outcome was significant (*t* = −2.37, *p* = 0.024, *η*^2^ = 0.16), and this interaction was mainly driven by the main effect of stimulation in the gain condition (*t* = −2.07, *p* = 0.046, *η*^2^ = 0.13; higher arousal ratings after inhibitory stimulation), while the respective main effect of stimulation in the loss condition was insignificant (*t* = 0.38, *p* = 0.694).

To investigate the framing effect, we also analyzed the SAM-arousal ratings in the “keep” condition only. In this analysis, only the main effect of outcome (gain, loss, i.e., here: frame) was significant (*t* = −4.04, *p* < 0.001, *η*^2^ = 0.36). Participants rated gain-framed outcomes (*M* = 3.23) as less arousing than loss-framed outcomes (*M* = 3.99), even though they both held the same value.

**Figure 8:**
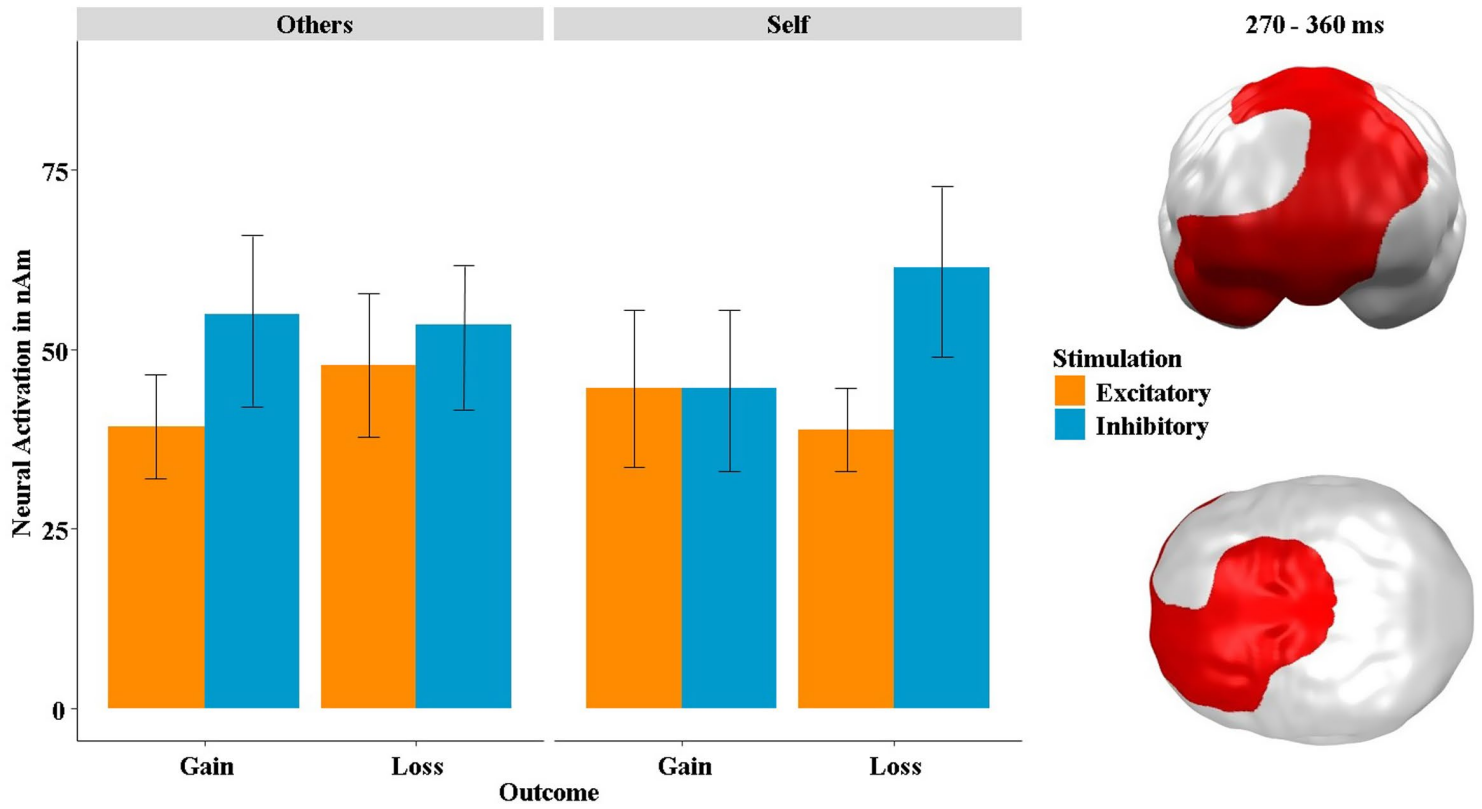
Significant spatiotemporal cluster in vmPFC, right OFC and dorsal prefrontal areas featuring a three-way interaction of stimulation × outcome × recipient. Bars indicate mean and 95% confidence intervals. Topographies of effects observed in L2-MNE were projected on standard 3D brain models for visualization.

At the neural level, we found a significant spatiotemporal cluster overlapping the vmPFC, right OFC and dorsal prefrontal areas, showing a three-way interaction of stimulation by outcome by recipient in a mid-latency time interval between 270 and 360 ms (*p*-cluster = 0.035; see Figure 8). *Post hoc* tests revealed that the stimulation by outcome interaction was significant within the “self” condition (*t* = 2.12, *p* = 0.045, *η*^2^ = 0.18) and also, at least by trend, in the “others” condition (*t* = −1.97, *p* = 0.061, *η*^2^ = 0.16). In the “others” condition, this interaction was mainly driven by differential effects induced by the stimulation after gains (*t* = −2.01, *p* = 0.042, *η*^2^ = 0.10), while in the “self” condition differential effects due to the stimulation were mainly observed after losses (*t* = −2.94, *p* = 0.004, *η*^2^ = 0.13). Since the same three-way interaction of stimulation by outcome by recipient was also present in the SAM-pleasantness rating (see Figure 7), we calculated a correlation between the neural and behavioral data, which revealed a highly significant positive correlation between the neural activity in this cluster and the SAM-pleasantness rating [*r*(382) = 0.21, *p* < 0.001]. The neural activity in this cluster was not correlated with the SAM-arousal rating [*r*(382) = 0.01, *p* = 0.823].

**Figure 9:**
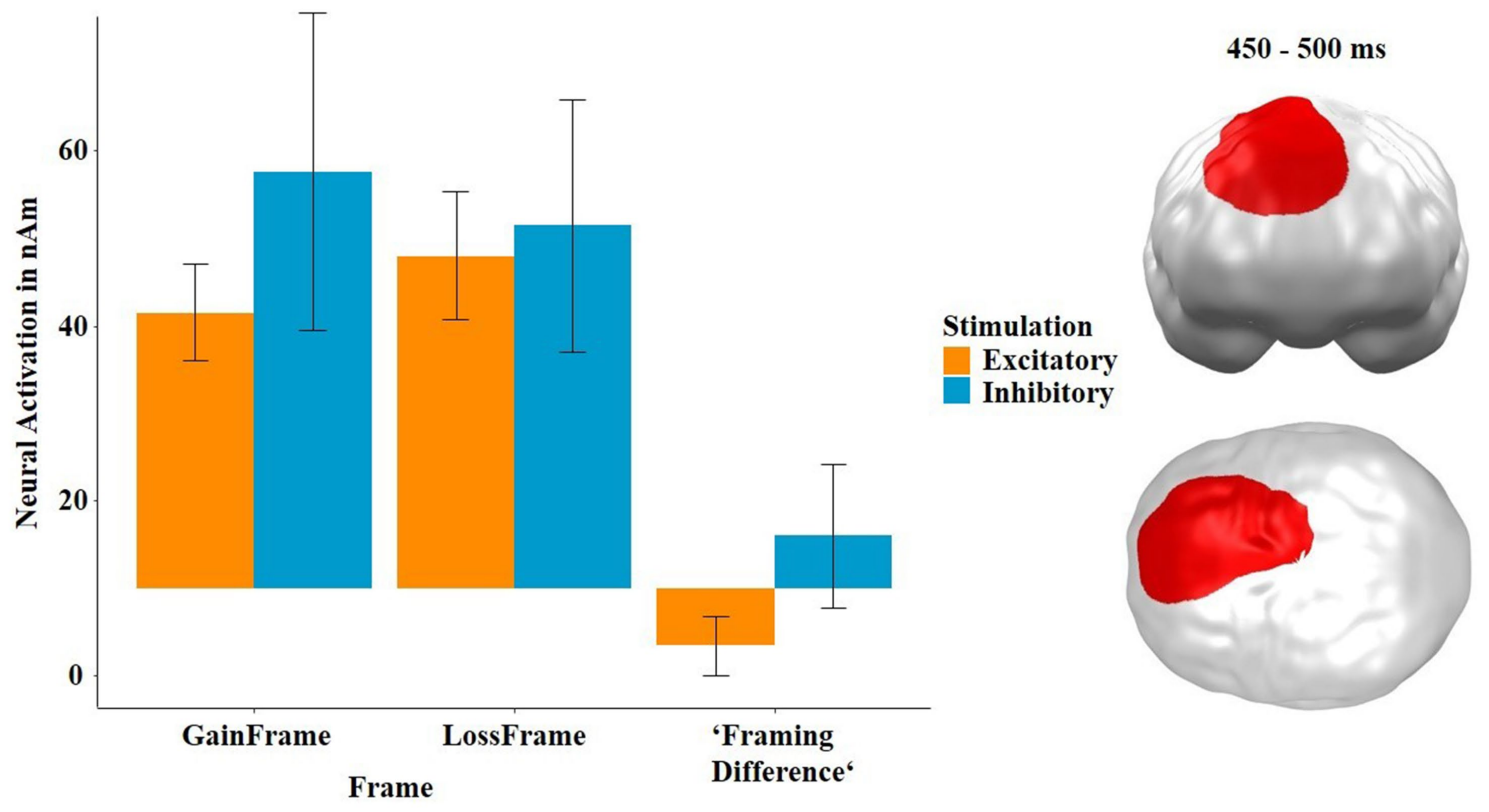
Significant spatiotemporal cluster in dorsomedial frontal areas featuring an interaction of stimulation × frame in the framing-relevant “keep” condition. Bars indicate mean and 95% confidence intervals. Topographies of effects observed in L2-MNE were projected on standard 3D brain models for visualization.

As for the behavioral data, we analyzed the neural data again in the “keep” condition only, because of our special interest in the framing effect, and used the predictors stimulation (excitatory, inhibitory), outcome (gain, loss, i.e., here: frame) and recipient (others, self). This analysis revealed two significant spatiotemporal clusters showing interactions with the factor stimulation (see Figure 9): first, we could replicate a cluster in dorsomedial frontal areas from our previous studies between 270 and 360 ms featuring an interaction effect of stimulation by frame (*p*-cluster = 0.028). As in our previous studies (Kroker et al., 2022, 2023a, 2023b), the neural framing difference in this cluster was smaller after excitatory than after inhibitory stimulation (*t* = −4.04, *p* < 0.004, *η*^2^ = 0.45). Moreover, and importantly, the neural framing difference in this cluster was strongly correlated with the behavioral framing difference in the SAM-pleasantness rating [*r*(382) = 0.22, *p* < 0.001], i.e., the activity in this cluster was strongly associated with actual rational gambling behavior, as reflected in the ratings. The neural framing difference in this cluster was not correlated with the framing difference in the SAM-arousal ratings [*r*(382) = −0.02, *p* = 0.858].

Second, we found a spatiotemporal cluster in dorsomedial, parietal and frontal areas at 270–360 ms showing a three-way interaction between stimulation, frame and recipient at 310 and 430 ms (*p*-cluster = 0.025). The *post hoc* tests indicated that the interaction was driven by both the interaction between stimulation and recipient within the gain frame (*t* = −3.74, *p* < 0.001, *η*^2^ = 0.36) and, though by trend, also within the loss frame (*t* = 1.93, *p* = 0.061, *η*^2^ = 0.13). Interestingly, the neural activity in this cluster also strongly correlated with the SAM-pleasantness ratings, but now, in contrast to the slightly earlier and more frontal cluster depicted in Figure 8, in a negative direction [*r*(382) = −0.20, *p* = 0.007]. Further *post hoc* tests on the difference (gain frame minus loss frame) revealed that this interaction was mainly driven by the main effect of stimulation in the “others” condition (*t* = 2.53, *p* = 0.019, *η*^2^ = 0.23), while the respective effect was not significant in the “self” condition (*t* = −1.57, *p* = 0.130). The behavioral and neural framing differences in this cluster were not correlated [pleasantness: *r*(382) = −0.08, *p* = 0.447; arousal: *r*(382) = 0.01, *p* = 0.892].

## Discussion

We investigated whether non-invasive stimulation of the vmPFC, a structure with cardinal functions for reward processing, modulates gambling behavior depending on whether one is playing for oneself or someone else. Using tDCS to either excite or inhibit the vmPFC, we replicated our previous finding that excitatory vmPFC stimulation (compared to inhibitory stimulation) resulted in improved risk weighing and, consequently, in more adaptive gambling behavior (see Figure 4). However, in contrast to our previous findings, this effect now occurred in both frames alike and not specifically in the loss frame. Interestingly, we also replicated our finding that modulated risk weighing was driven by altered affective learning following vmPFC excitation (see Figure 5B). However, in the current study this effect only occurred when participants played for themselves but not when they played for someone else. The neural data suggest that excitation of the vmPFC results in increased neural activity relative to vmPFC-inhibition when playing for oneself compared to playing for someone else in the decision phase. In the feedback phase (i.e., when gain or loss icons were presented), we found that the stimulation predominately modulated the perceived (un)pleasantness of loss feedback (see Figure 7). Losses were rated more negatively when they affected oneself versus someone else (i.e., participants were more sensitive to their own losses), and this tendency of enhanced loss sensitivity was rather amplified by excitatory compared to inhibitory stimulation. At the neural level (see Figure 8), excitatory stimulation reduced neural activity at the vmPFC and neighboring PFC regions also particularly for losses in the self-referencing condition. This nicely converged with the behavioral findings, supported by the highly significant positive correlation between neural activity and the SAM-pleasantness ratings. We further replicated findings showing that a cluster in prefrontal regions reflect the neural framing effect depending on the stimulation, and, importantly, the framing effect within this cluster was correlated with the behavioral framing effect. Finally, we also identified a neural cluster suggesting that the neural framing depending on the stimulation is also modulated by the recipient.

At the behavioral level, we were unable to replicate our prior finding that the framing effect is modulated by the stimulation in the decision phase (see Figure 4). This could be due to the cumulative effects of stimulation and recipient, as participants often display more rational gambling behavior when playing for others, i.e., a smaller framing difference. In line with this explanation, prior work has indicated that people evaluate gambling more rationally when they are not affected themselves (Youn et al., 2000). In addition, stimulation might also modulate gambling behavior, although none of the two mentioned effects reached significance in the decision phase. Furthermore, simply adding another factor and its associated main effects adds extra variance in the statistical model, so this could also explain why we could not replicate the interaction effect of stimulation by frame in the decision phase. Additionally, in this study the general effect of inhibitory stimulation independent of frame or recipient, which increases willingness to take risks, was stronger than in our previous within-design study (Kroker et al., 2022). This might also mask the interaction effect of stimulation and frame.

Importantly, as found in many previous studies, here we also found an interaction between stimulation and risk of losing (see Figure 5A), whereby more adaptive gambling behavior occurred after excitatory versus inhibitory stimulation (Kroker et al., 2022, 2023a, 2023b; Rehbein et al., 2023). This altered rationality is characterized by a greater tendency to gamble when the chance of winning is high and a greater tendency to avoid risk when the risk of losing is high. Interestingly, although unexpected, excitatory stimulation not only relatively increased rationality for self-referenced gambling, but it also increased rationality for other-referenced gambling, which was significant, albeit somewhat weaker. However, the three-way interaction between stimulation, risk of losing and trial number (i.e., progress of learning) was modulated by recipient; i.e., the four-way interaction of stimulation by risk of losing by recipient by trial number was significant. *Post hoc* tests revealed that participants showed relatively improved learning over the paradigm after excitatory stimulation only when playing for themselves (see Figure 5B). As an explanation, other researchers have suggested that participants use the information they receive when they gamble for others to maximize their own gains (Yu and Zhou, 2006), and this could be promoted by excitatory stimulation. However, the literature on this topic is thin. As shown in Supplementary Figure 1, the stimulation order influences choice behavior and the resulting actual wins. This indicates that, compared to inhibitory stimulation, excitatory stimulation not only induces facilitated learning from the beginning of the experiment, but also carry-over effects from the previous stimulation. Possibly excitatory stimulation relatively improves the reactivation of gambling skills learned in the first (inhibitory) session, while inhibitory stimulation impairs this ability. However, this does not alter the conclusion that vmPFC stimulation improves overall choice behavior and learning relative to inhibitory stimulation, it merely changes the underlying (learning) pattern.

This behavioral finding is particularly interesting in light of the neural results. Specifically, the interaction cluster of stimulation by recipient in the left ventral prefrontal and anterior temporal areas (80–120 ms) indicates greater neural activity in the excitatory condition when participants played for themselves (see Figure 6). This could be interpreted to mean that participants put more effort into maximizing their own outcomes when they have a more active vmPFC. Ultimately, this could have resulted in improved learning in the excitatory condition compared to inhibitory stimulation. Furthermore, the correlations in this cluster are very interesting, since ventral prefrontal activity is typically associated with the inhibition of maladaptive behavioral responses not only in gambling (van Holst et al., 2010a, 2010b) but also, for example, in fear paradigms (Kaczkurkin et al., 2017; Lissek et al., 2014). Thus, one would expect a negative correlation between the neural activity in this cluster and risk-taking behavior. However, this seems to strongly depend on the condition, as the correlation with risky choices was negative in the excitatory and “others” conditions and positive in the inhibitory and “self” conditions. Accordingly, other higher cognitive functions and not just behavioral inhibition must also play a role here. For example, van Holst et al. (2012) showed that the vmPFC is also involved in reward anticipation, as is reflected in the inhibitory and “self” condition, which are rather associated with irrational behavior in the present study. On the other hand, the excitatory condition and the “others” condition could trigger the inhibitory function of this ventral prefrontal area (Hiser and Koenigs, 2018).

**Figure 10:**
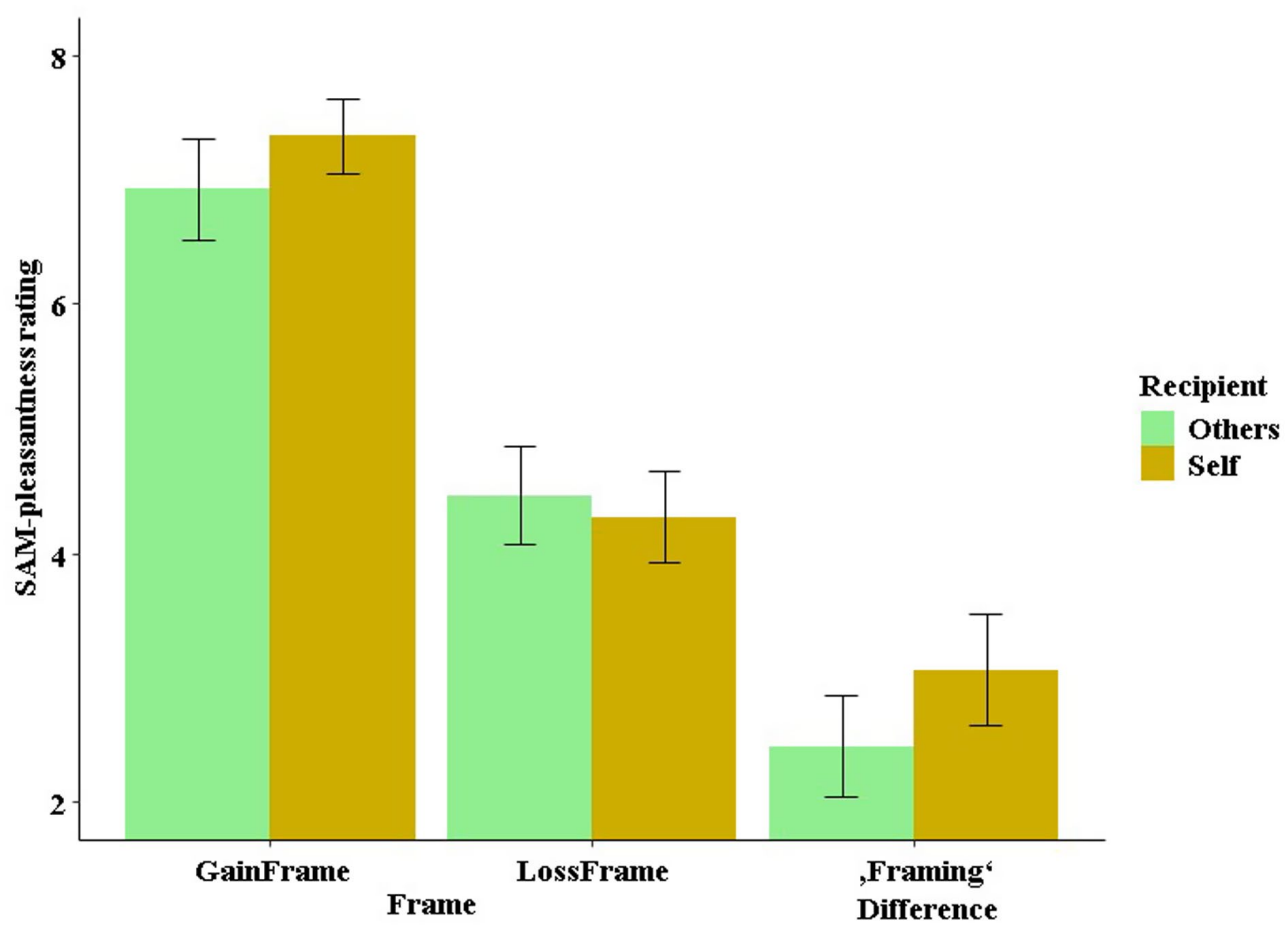
SAM-pleasantness rating in the “keep” condition as a function of frame and recipient (self vs. other). Here, a stronger framing effect (i.e., a stronger framing difference between the gain and loss frames) was observed when playing for oneself than when playing for others.

In the feedback phase, we found a three-way interaction of stimulation by outcome by recipient affecting the SAM-pleasantness ratings, which was particularly driven by the interaction of stimulation by recipient in the loss condition (see Figure 7). Participants rated losses more negatively after excitatory stimulation when they played for themselves, while this effect was inverted after inhibitory stimulation. The more negative rating of losses may indicate an enhanced processing depth, which could ultimately result in comparatively improved learning from losses after excitatory stimulation when playing for oneself. In this regard, researchers have already proposed that avoiding losses or risks was important for evolutionary survival and is, nowadays, important for economic sustainability (Gigerenzer, 2018; Todd and Gigerenzer, 2000). Thus, excitatory stimulation could help us avoid unreasonable risks. Furthermore, we tested whether stimulation and recipient influenced the SAM-pleasantness ratings in the framing-relevant “keep” condition, and we did in fact reveal an interaction between frame and recipient (see Figure 10). This indicates a reduced framing difference (gain frame rating − loss frame rating), i.e., more rational ratings, when participants gambled for others compared to when participants gambled for themselves. This makes sense, since gambling for others does not require a more cautious avoidance of risk (i.e., loss aversion) (Kahneman and Tversky, 1979), as one’s own assets are not at risk.

**Figure 11:**
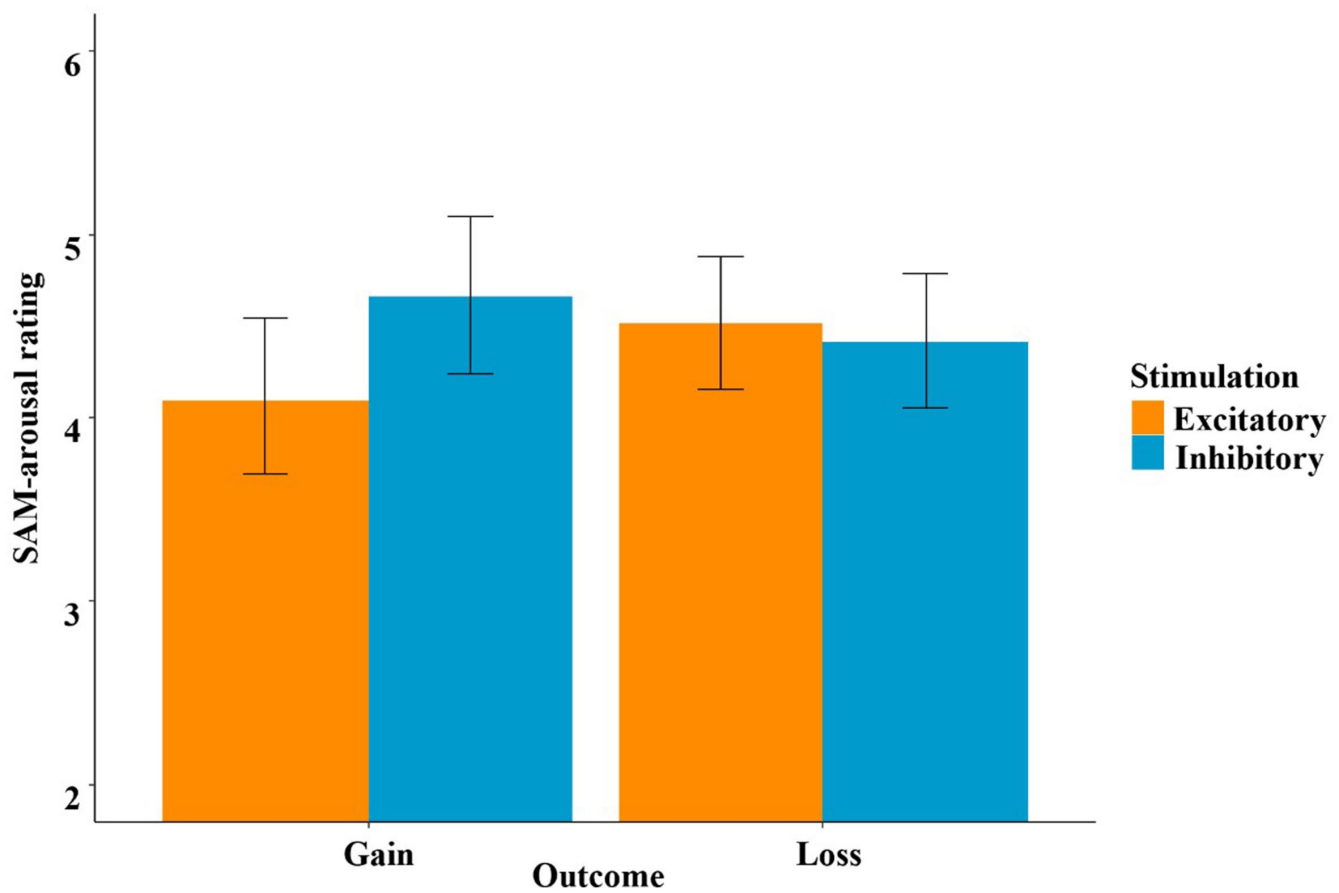
SAM-arousal ratings as a function of stimulation and outcome. This indicates higher arousal ratings for gains after inhibitory stimulation.

In the SAM-arousal ratings, we observed an interaction effect of stimulation and outcome, which was strongly driven by greater arousal after gains in the inhibitory condition compared to the excitatory condition (see Figure 11). This might be interpreted as an overweighing of short-term gains, resulting in a poor and short-sighted strategy. This is typically seen in patients with vmPFC lesions or hypoactivity, such as pathological gamblers (Antons et al., 2020; van Holst et al., 2010a, 2010b), who fail to disengage from gambling (Bechara et al., 2000; Perales et al., 2020).

At the neural level, we observed the same three-way interaction of stimulation by outcome by recipient, which we also found in the behavioral data. The respective cluster was located in prefrontal and parietal areas at 270–360 ms and overlapped the vmPFC (see Figure 8). This was mainly driven by effects of stimulation on the processing of gains in the “others” condition and the processing of losses in the “self” condition. After excitatory stimulation, gains and losses made for others were less strongly processed. Again, this suggests that the vmPFC is particularly involved in maximizing one’s own benefit, which makes sense, as the vmPFC is also part of the reward system (Arias-Carrián et al., 2010). This is supported by the finding that the neural activity in this cluster is positively correlated with SAM-pleasantness ratings, suggesting that this cluster may be involved in particularly positive ratings after gains. Furthermore, this correlation supports the assumption that the vmPFC and more dorsal prefrontal regions are associated with higher evaluative processes such as feedback evaluation. Analogous to the behavioral data, we computed a further analysis in the “keep” condition only. This revealed an interaction effect in dorsomedial prefrontal areas between 450 and 500 ms (see Figure 9) and, again, represents a replication of our earlier findings (Kroker et al., 2022, 2023a, 2023b), which showed the exact same pattern. Importantly, the positive correlation between the neural and the behavioral framing differences suggests that the activity in this cluster is actually associated with irrational feedback processing, because the greater the framing effect/difference in this cluster, the greater the behavioral framing effect/difference. This is puzzling at a first glance, as one would rather expect a negative correlation of the framing effect with this dorsomedial prefrontal area. However, the dorsomedial prefrontal cortex is part of the task-positive network, whereas the ventral prefrontal cortex is part of the default-mode network (Cheng et al., 2020) typically showing an anti-correlation (Fox et al., 2006). Thus, a correlation with the framing effect within ventral prefrontal areas should have been expected to be negative, which has been shown previously (DeMartino et al., 2006).

**Figure 12:**
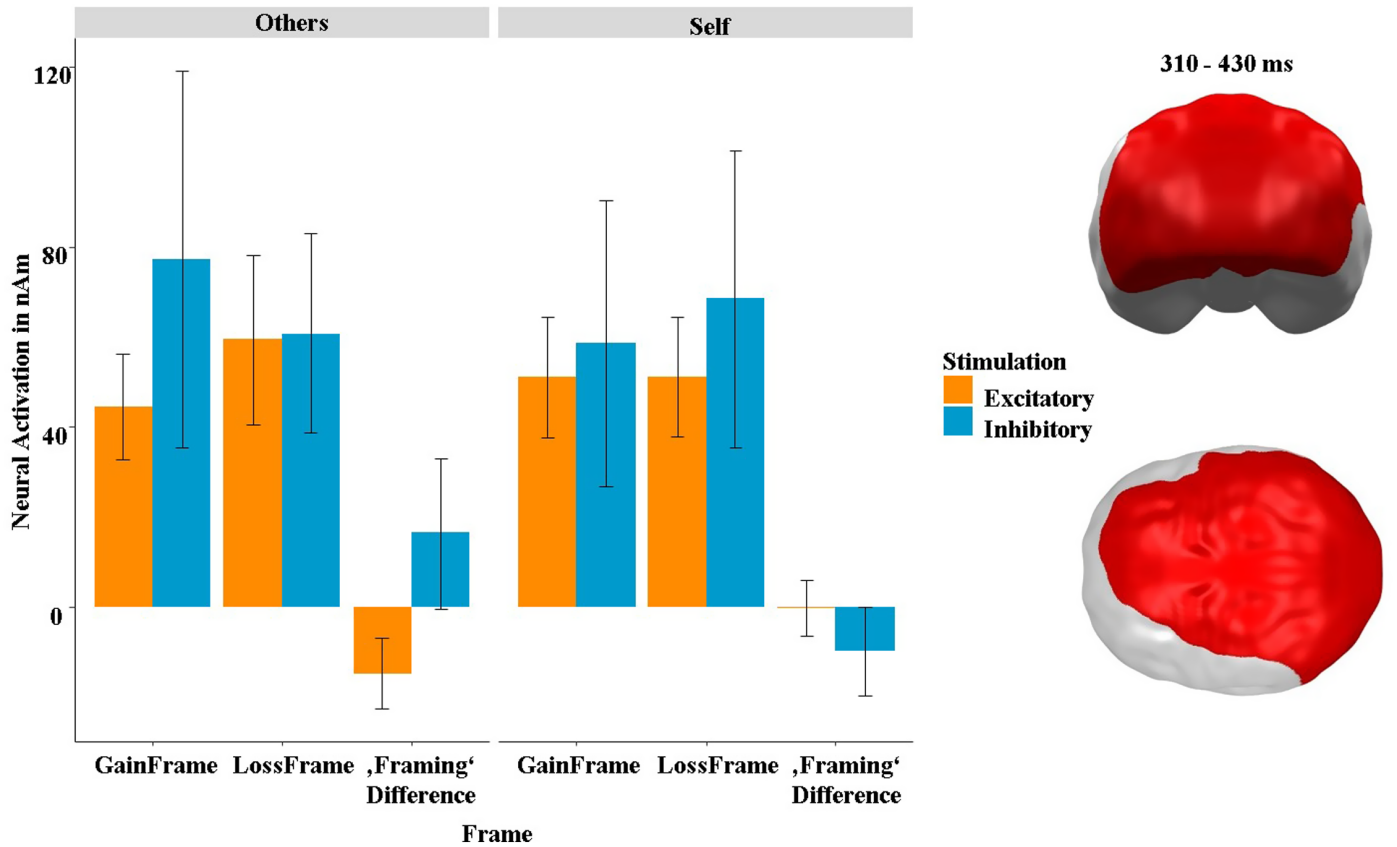
Significant spatiotemporal cluster in dorsomedial frontal, parietal and frontal areas featuring a three-way interaction of stimulation × frame × recipient in the framing-relevant “keep” condition. “Framing Difference” refers to the difference of GainFrame minus LossFrame. Bars indicate mean and 95% confidence intervals. Topographies of effects observed in L2-MNE were projected on standard 3D brain models for visualization.

Finally, we observed a large rather posterior cluster, which also reaches into frontal areas at 310 to 430 ms (Figure 12). This cluster showed an attenuated neural framing effect/difference after excitatory stimulation in the “self” condition, which was not present in the inhibitory condition. This would indicate that excitatory stimulation relatively weakens the tendency that participants gamble more rationally when they play for others (Youn et al., 2000), as vmPFC excitation primarily enhances gambling when playing for oneself (Figure 5). However, this interpretation is highly speculative, and further research is necessary to clarify this research question. Unfortunately, this cluster was not correlated with the behavioral data, further complicating the interpretation of this neural effect.

### Limitations and implications for future research

Although our study has provided novel insights into the potential causal role of the vmPFC in gambling and self-and other-referencing, several aspects and limitations require consideration. Crucially, the stimulation montage utilized in this study also co-stimulated, though to a smaller degree, other prefrontal regions implicated in self-and other-referencing in gambling. To address this limitation, alternative stimulation methods, such as rTMS, could be employed to target focal prefrontal regions with greater precision. Furthermore, we did not use a sham condition in this study limiting the interpretability regarding clinical applications of vmPFC-tDCS. However, we have shown that the sham condition is typically “in between” the excitatory and inhibitory condition (Kroker et al., 2025; Roesmann et al., 2021). Accordingly, the interpretations regarding causal functionality of the vmPFC remain unaffected. Notably, we applied this within-subjects design to reduce interindividual variance and successfully blind participants to the experimental conditions, as participants typically recognize the difference between active and sham stimulation, but not between the two active conditions. In addition, we must acknowledge that our sample consisted mainly of young adults, so we cannot generalize our findings to the general population. This is particularly important when considering the first and second onset peaks of pathological gambling, which are 15–19 and 40–44 years of age, respectively (Black et al., 2015). Thus, the neurocognitive risk factors identified here do not fully apply to these age groups. Furthermore, we were unable to replicate the behavioral framing effect in the decision and feedback phases. This interaction was not present in the “self” nor in the “others” conditions, suggesting that it was not dependent on the recipient. Future studies may be able to determine whether this was due to additional variance that was added by the factor recipient, was due to the stronger effects of the inhibitory stimulation in this study or was caused by something else. Interpreting three-way interactions involving the recipient at the neural level is challenging (Figures 8, 12), particularly in the absence of a corresponding behavioral finding or correlation with behavioral data, as the results in both conditions could always be interpreted in both directions. In general, we have to acknowledge the fact that we have observed dissociations between behavioral and neural data. It is possible that vmPFC stimulation modulates subtle aspects of neural processing that contribute to behavior, but where other factors (e.g., individual variability, task complexity/engagement, or the strength of other competing neural processes) might dilute the behavioral effect of interest in our paradigm. Furthermore, it is well known that the sensitivity of MEG sensors is lower in prefrontal areas (Coquelet et al., 2020), so that these neural effects must be evaluated more cautiously. Another aspect to consider is that we did not gather ratings right after the trials, but only at the end of the experiment. This may have induced recency effects and thus biased the ratings, so that the last stimuli influenced the ratings to greater degree (Di Plinio et al., 2020). Future studies should look at the stimulation effects in more detail, which could be done by including demographic und personality data as covariates or use individualized stimulation montages. This would not only optimize the stimulation (i.e., stimulation of the intended target), but also reveal whether the stimulation has stronger effects on certain groups. In addition, it should be considered that participants played for other participants (i.e., strangers), whereas gambling behavior might be different if they had played for a person with whom they have a close bond. Therefore, future studies should use paradigms in which participants play for a person with whom they have a closer relationship. Because fMRI studies have shown that feeling empathy for a friend activates the mPFC, while feeling empathy for a stranger does not (Meyer et al., 2013, 2015). This could be, because feeling empathy for friend may be more related to the default-mode network, to which the vmPFC belongs and is known to be rather associated with emotional processing (Cheng et al., 2020). Feeling empathy for a stranger, on the other hand, possibly activates the “cold” or “more cognitive” task-positive network in more dorsal prefrontal regions. These could be more involved in the rather cognitive aspects of perspective-taking. Furthermore, it could be that feeling empathy for a friend, goes along with a greater benefit for the individual playing, and thus activates the vmPFC.

## Conclusion

In summary, the present study suggests that non-invasive stimulation of the vmPFC modulates gambling depending on who the gambling will affect (self or others). The behavioral and neural results suggest that excitation (compared to inhibition) of the vmPFC increases the depth of processing and the effort to maximize one’s own gains, whereas this effect is small or absent in the “others” condition. As such, the participants’ learning process was modulated by the stimulation as a function of whom they were playing for. This effect was supported by the different correlations between risk-taking behavior and neural activity in ventral prefrontal areas for the “self” and “others” conditions. This provides evidence for our hypothesis predicting comparatively enhanced gambling predominantly in the “self” condition. In the feedback phase, participants were less susceptible to the framing effect when playing for someone else than when playing for themselves, while stimulation did not affect the framing effect, as we would have expected. The neural effects, however, suggest that the framing effect is differentially affected by the stimulation between the “self” and “others” conditions, which could indicate that participants process the framing more deeply when playing for themselves, as they are more susceptible to the effect in this condition. In summary, our results may indicate improved gambling and learning from feedback across paradigms following excitatory stimulation at the behavioral and neural levels. The novel finding of this study is the differential modulation of gambling behavior by vmPFC-tDCS in a recipient-dependent manner. This is evidenced by opposite interaction patterns at the neural level (see Figure 12), different learning patterns at the behavioral level (see Figure 5), and notably different correlations between recipients in prefrontal clusters that were modulated by the stimulation. These results clearly indicate that gambling performance is enhanced after excitatory compared to inhibitory stimulation when playing for oneself, but not when playing for others. Thus, our findings support more complex neurocognitive models of empathy, theory of mind, and self-referencing that posit interactions between these processes, but also certain differences.

## Data Availability

The raw data supporting the conclusions of this article will be made available by the authors, without undue reservation.
