# Supplementary material for: Self-referencing versus other-referencing in gambling: effects of vmPFC stimulation on decision-making and feedback processing
Source: Front Behav Neurosci. 2025 Sep 9;19:1634058. doi: 10.3389/fnbeh.2025.1634058 (PMC12454093; doi:10.3389/fnbeh.2025.1634058)
Supplement: Supplementary file 1 [file Data_Sheet_1.docx]

**Supplementary Materials**

**Self-Referencing vs. Other-Referencing in Gambling: Effects of vmPFC Stimulation on Decision making and Feedback Processing**

**Thomas Kroker, PhD^1,2^, Maimu Alissa Rehbein, PhD^1,2,4^, Miroslaw Wyczesany, PhD^3^, Riccardo Bianco, MSc^1,2^, Alejandro Espino-Paya, PhD^1,2^, Selina Teresa Hansen, MD^1,5^ & Markus Junghöfer, PhD^1,2^**

^1^ Institute for Biomagnetism and Biosignalanalysis, University of Muenster, Germany

^2^ Otto Creutzfeldt Center for Cognitive and Behavioral Neuroscience, University of Muenster, Muenster, Germany

^3^ Institute of Psychology, Jagiellonian University, Krakow, Poland

^4^ Institute of Psychology, Unit of Clinical Psychology and Psychotherapy for Children and Adolescents, University of Osnabrueck

^5^ Department of Child and Adolescent Psychiatry, University Hospital Muenster, Muenster, Germany

Corresponding author:

Thomas Kroker

Institute for Biomagnetism and Biosignalanalysis

University of Münster

Malmedyweg 15

48149 Münster, Germany

Email: thomas.kroker@uni-muenster.de

**Funding**: Supported by the DFG (project JU 445/9-1), the National Science Center (UMO-2018/31/G/HS6/02490) and the Clinician Science Program (CareerS) of the Muenster University Clinic to SH.

**Methods**

**1.1 Participants**

**Table 1**

Demographic and psychometric characteristics of participants

|  | **M/N** | **SD** | | | ***χ²*** | | | **df** | **p** | |  |  | | | | | | |
| --- | --- | --- | --- | --- | --- | --- | --- | --- | --- | --- | --- | --- | --- | --- | --- | --- | --- | --- |
| **Demographic**  **Characteristic** |  | |  |  | |  |  | | |  | | |  | |  | | |  |
| N | 32 |  | | |  | | |  |  | | | | |  | |  |  |  |
| Female (N, %) | 14 (43.75) | - | | | 0.5 | | | 1 | 0.479 | | | | |  | |  |  |  |
| Age (years) | 24.09 | 3.60 | | | - | | | - | - | | | | |  | |  |  |  |
| Stimulation order  (Exc-Inh, %) | 17 | - | | | 0.12 | | | 1 | 0.724 | | | | |  | |  |  |  |
| **Psychometric**  **Characteristics** |  |  | | |  | | |  |  | | | | |  | |  |  |  |
| BDI-II | 3.18 | 2.10 | | |  | | |  |  | | | | |  | |  |  |  |
| RR | 24.67 | 3.07 | | |  | | |  |  | | | | |  | |  |  |  |
| UI-18 | 43.47 | 8.80 | | |  | | |  |  | | | | |  | |  |  |  |
| SDS | 13.42 | 4.35 | | |  | | |  |  | | | | |  | |  |  |  |
| PANAS-Pos-Exc | 25.31 | 7.04 | | |  | | |  |  | | | | |  | |  |  |  |
| PANAS-Pos-Inh | 24.94 | 7.41 | | |  | | |  |  | | | | |  | |  |  |  |
| PANAS-Neg-Exc | 13.12 | 6.21 | | |  | | |  |  | | | | |  | |  |  |  |
| PANAS-Neg-Inh | 12.86 | 6.73 | | |  | | |  |  | | | | |  | |  |  |  |

*Note.* Stimulation order – excitatory first, inhibitory second: N = 17; inhibitory first, excitatory second: N = 15. BDI-II = Beck Depression Inventory (Beck et al., 1996). RR = Reward responsiveness scale (Van den Berg et al., 2010). UI-18 = Intolerance of Uncertainty scale (Gerlach et al., 2008). SDS-CM = Social desirability scale (Crowne & Marlowe, 1960). PANAS = Positive and negative affective schedule (Watson et al., 1988).

**Results and discussion**

## **2.1. Decision-making phase**

As indicated in the main text, the control analysis including stimulation order revealed significant effects on the interactions of stimulation by risk of losing (i.e., here threeway interaction; z = 4.55, p < 0.001, OR = 1.99) and stimulation by risk of losing by trial number (i.e., here fourway interaction; *z* = -3.42, *p* < 0.001, *OR* = 0.61).


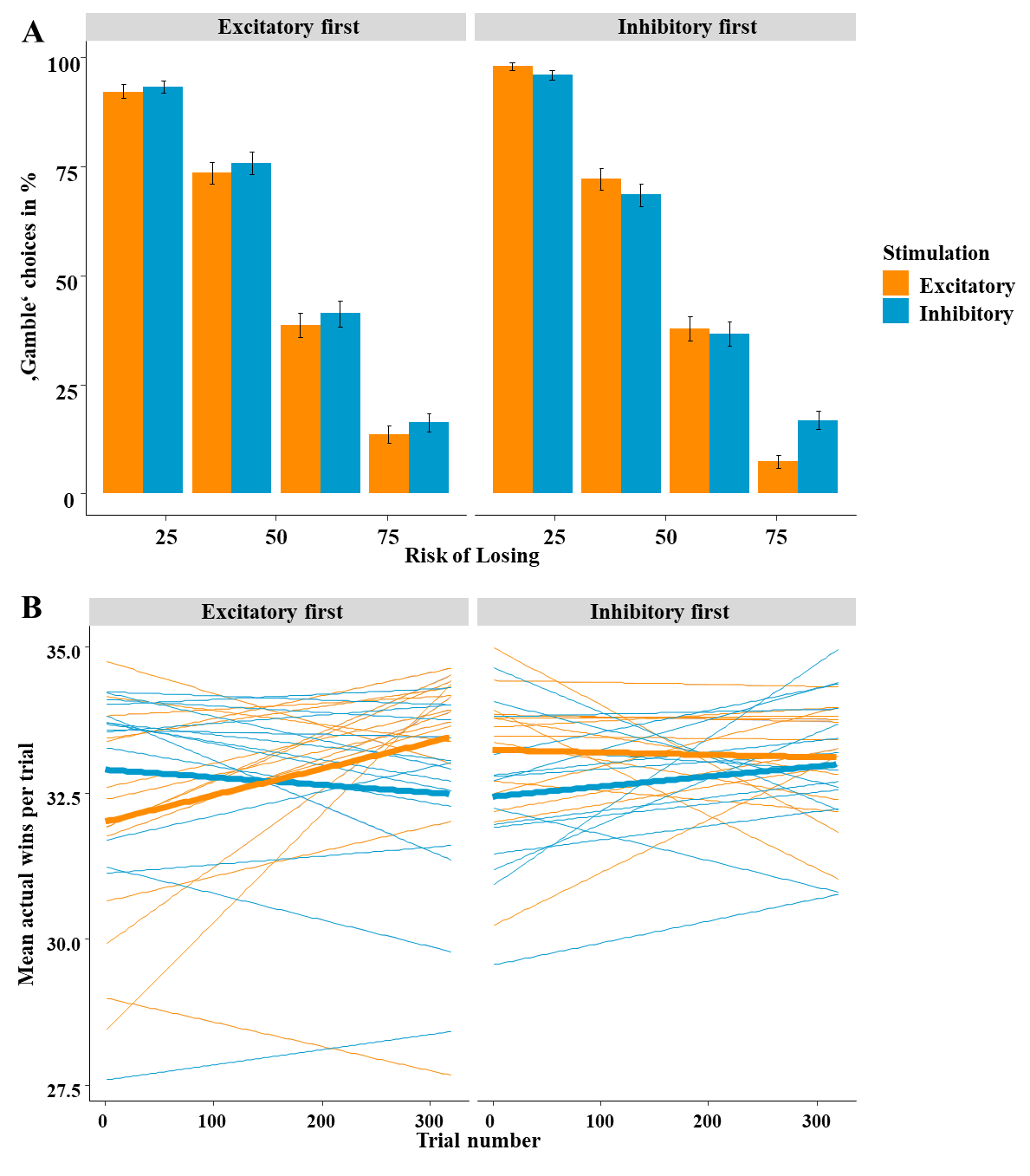


*Figure SM1.* **A.** Significant threeway interaction of stimulation, risk of losing and stimulation order. **B.** Fourway interaction of stimulation, risk of losing, trial number and stimulation order, resulting in a different learning pattern as a function of which stimulation was conducted first.

The 2x2x2 repeated-measures ANOVA with the factors stimulation (excitatory, inhibitory), frame (gain-frame, loss-frame) and recipient (others, self) revealed a main effect of recipient. The cluster occurred at 20 to 80 ms in right prefrontal areas displaying a higher neural activation in response to the others- compared to self-condition (*p*-cluster = 0.036). This cluster may reflect that more effort is required when playing for someone else, because participants first have to get used to playing for someone else. For which self-referencing may be a necessary condition at the neural level (Jenkins et al., 2008).


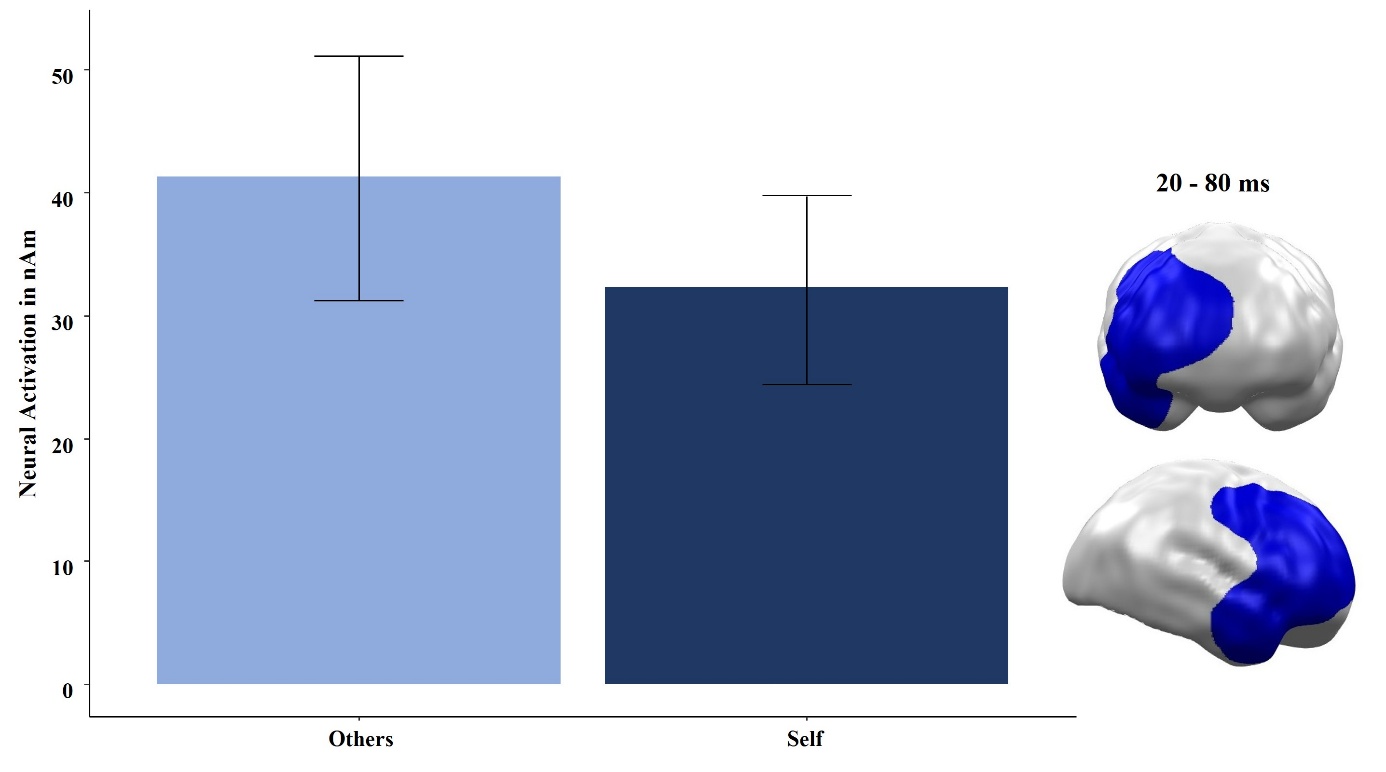
 *Figure SM2.* Significant spatiotemporal cluster in right prefrontal and anterior temporal areas featuring a main effect of recipient (others vs. self).

Bars indicate mean and 95% confidence intervals. Topographies of effects observed in L2-MNE were projected on standard 3D brain models for visualization.

## **2.2. Feedback phase**

The 2x2x2x2 repeated-measures ANOVA with the factors stimulation (excitatory, inhibitory), decision (keep, gamble), outcome (gain, loss) and recipient (others, self) showed a cluster with a main effect of outcome. The cluster occurred at 0 to 400 ms and covers the whole brain (*p*-cluster = 0.008). This cluster shows a greater activity in response to losses compared to gains. This cluster is most probably due to arousal effects as losses were typically rated as more arousing than gains, which has already been described in the influential prospect theory (Kahneman & Tversky, 1979; Tversky & Kahneman, 1992). Furthermore, we found similar clusters already in our previous studies (Kroker et al., 2022, 2023; Rehbein et al., 2023).


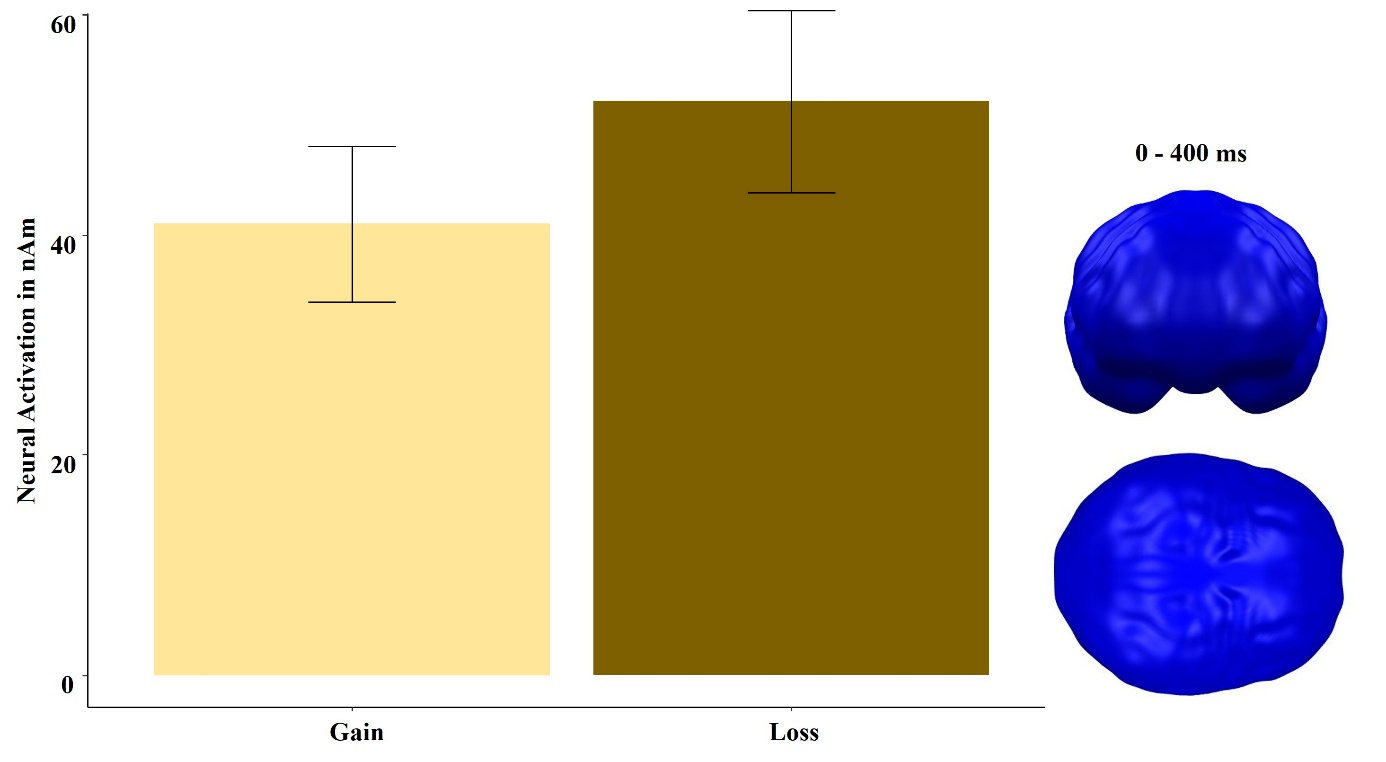
 *Figure SM3.* Significant spatiotemporal cluster covering the whole brain featuring a main effect of outcome (gain vs. loss).

Bars indicate mean and 95% confidence intervals. Topographies of effects observed in L2-MNE were projected on standard 3D brain models for visualization.

**References**

Beck, A., Steer, R., & Brown, G. (1996). *Manual for the Beck Depression Inventory-II*. TX Psychological Corporation.

Crowne, D. P., & Marlowe, D. (1960). A new scale of social desirability independent of psychopathology. *Journal of Consulting Psychology*, *24*(4), 349–354. https://doi.org/10.1037/h0047358

Gerlach, A. L., Andor, T., & Patzelt, J. (2008). Die bedeutung von unsicherheits-intoleranz für die generalisierte angststörung: Modellüberlegungen und Entwicklung einer deutschen version der unsicherheitsintoleranz-skala. *Zeitschrift Fur Klinische Psychologie Und Psychotherapie*, *37*(3), 190–199. https://doi.org/10.1026/1616-3443.37.3.190

Jenkins, A. C., Macrae, C. N., & Mitchell, J. P. (2008). *Repetition suppression of ventromedial prefrontal activity during judgments of self and others*. www.pnas.org/cgi/content/full/

Kahneman, D., & Tversky, A. (1979). Prospect Theory: An Analysis of Decision under Risk. *Econometrica*, *47*(2), 263–291. https://econpapers.repec.org/RePEc:ecm:emetrp:v:47:y:1979:i:2:p:263-91

Kroker, T., Wyczesany, M., Rehbein, M. A., Roesmann, K., Wessing, I., & Junghöfer, M. (2022). Noninvasive stimulation of the ventromedial prefrontal cortex modulates rationality of human decision-making. *Scientific Reports*, *12*(1), 20213. https://doi.org/10.1038/s41598-022-24526-6

Kroker, T., Wyczesany, M., Rehbein, M. A., Roesmann, K., Wessing, I., Wiegand, A., Bölte, J., & Junghöfer, M. (2023). Excitatory stimulation of the ventromedial prefrontal cortex reduces cognitive gambling biases via improved feedback learning. *Scientific Reports*, 1–17. https://doi.org/10.1038/s41598-023-43264-x

Rehbein, M. A., Kroker, T., Winker, C., Ziehfreund, L., Reschke, A., Bölte, J., Wyczesany, M., Roesmann, K., Wessing, I., & Junghöfer, M. (2023). Non-invasive stimulation reveals ventromedial prefrontal cortex function in reward prediction and reward processing. *Frontiers in Neuroscience*, *17*. https://doi.org/10.3389/fnins.2023.1219029

Tversky, A., & Kahneman, D. (1992). Advances in prospect theory: Cumulative representation of uncertainty. *Journal of Risk and Uncertainty*, *5*(4), 297–323. https://doi.org/10.1007/BF00122574

Van den Berg, I., Franken, I. H. A., & Muris, P. (2010). A new scale for measuring reward responsiveness. *Frontiers in Psychology*, *1*(DEC), 1–7. https://doi.org/10.3389/fpsyg.2010.00239

Watson, D., Clark, L. A., & Tellegen, A. (1988). Development and validation of brief measures of positive and negative affect: the  PANAS scales. *Journal of Personality and Social Psychology*, *54*(6), 1063–1070. https://doi.org/10.1037//0022-3514.54.6.1063
